# Mesenchymal stem cell transplantation in newly diagnosed type-1 diabetes patients: a phase I/II randomized placebo-controlled clinical trial

**DOI:** 10.1186/s13287-022-02941-w

**Published:** 2022-06-20

**Authors:** Mahmoud Izadi, Anavasadat Sadr Hashemi Nejad, Maedeh Moazenchi, Safdar Masoumi, Ali Rabbani, Farzad Kompani, Amir Abbas Hedayati Asl, Fatemeh Abbasi Kakroodi, Neda Jaroughi, Mohammad Ali Mohseni Meybodi, Aria Setoodeh, Farzaneh Abbasi, Seyedeh Esmat Hosseini, Fatemeh Moeini Nia, Reza Salman Yazdi, Roghayeh Navabi, Ensiyeh Hajizadeh-Saffar, Hossein Baharvand

**Affiliations:** 1 Department of Stem Cells and Developmental Biology, Cell Science Research Center, Royan Institute for Stem Cell Biology and Technology, ACECR, Tehran, Iran; 2 Advanced Therapy Medicinal Product Technology Development Center (ATMP-TDC), Cell Science Research Center, Royan Institute for Stem Cell Biology and Technology, ACECR, Tehran, Iran; 3 Department of Epidemiology and Biostatistics, Tehran University of Medical Sciences, Tehran, Iran; 4 Growth and Development Research Center, Children’s Medical Center of Excellence, Tehran University of Medical Sciences, Tehran, Iran; 5 Division of Hematology and Oncology, Children’s Medical Center, Pediatrics Center of Excellence, Tehran University of Medical Sciences, Tehran, Iran; 6 Department of Regenerative Medicine, Cell Science Research Center, Royan Institute for Stem Cell Biology and Technology, ACECR, Tehran, Iran; 7 Hematology-Oncology and Stem Cell Research Center, Shariati Hospital, Tehran University of Medical Sciences, Tehran, Iran; 8 Division of Pediatrics Endocrinology, Children’s Medical Center, Pediatrics Center of Excellence, Tehran University of Medical Sciences, Tehran, Iran; 9 Department of Medical-Surgical Nursing, School of Nursing and Midwifery, Iran University of Medical Sciences, Tehran, Iran; 10 Department of Andrology, Reproductive Biomedicine Research Center, Royan Institute for Reproductive Biomedicine, ACECR, Tehran, Iran; 11 Department of Diabetes, Obesity, and Metabolism, Cell Science Research Center, Royan Institute for Stem Cell Biology and Technology, ACECR, Tehran, Iran; 12 Department of Developmental Biology, School of Basic Sciences and Advanced Technologies in Biology, University of Science and Culture, Tehran, Iran

**Keywords:** Type 1 diabetes, Mesenchymal stem cells, Cell therapy, Immunomodulation, Randomized controlled trial, Regulatory T cells

## Abstract

**Background:**

Type-1 diabetes (T1D) occurs following autoimmune-induced pancreatic beta cells death. Among several treatment modalities, mesenchymal stem cells (MSCs) transplantation is promising for autoimmune disorders due to immunomodulation, regeneration, and migration to damaged tissue upon systemic injection. This study assessed the safety and efficacy of intravenous injection of autologous bone marrow-derived MSCs in newly diagnosed T1D patients.

**Methods:**

After receiving informed consent, 21 patients who met the study criteria were enrolled and randomly assigned to receive either MSCs or placebo. Each patient in the experimental group received two doses of MSCs and was followed for at least one-year post-transplantation.

**Results:**

The results have shown that this transplantation is safe and significantly reduces the number of hypoglycemic episodes. MSCs transplantation improved glycated hemoglobin (HbA1c), shifted serum cytokine patterns from pro-inflammatory to anti-inflammatory, increased the number of regulatory T-cells in the peripheral blood, and improved quality of life. Early transplantation of MSCs significantly improved HbA1c and C-peptide levels and shifted pro-inflammatory cytokines to anti-inflammatory cytokines. Also, exercise combined with MSCs transplantation improved glycemic and immunologic indices.

**Conclusions:**

Taken together, autologous MSC transplantation is safe and effective, and its early transplantation is a promising treatment in newly diagnosed T1D children suffering from hypoglycemic episodes.

*Trial registration*: This clinical trial was registered at the Iranian Registry of Clinical Trials (IRCT) with the identifier IRCT ID: IRCT2016070428786N1 registered on August 20, 2016 (Retrospectively registered) (https://en.irct.ir/trial/23256) and at the U.S. National Institutes of Health (ClinicalTrials.gov) with the related identifier NCT04078308 registered on September 6, 2019 (Retrospectively registered). (https://clinicaltrials.gov/ct2/show/NCT04078308).

**Supplementary Information:**

The online version contains supplementary material available at 10.1186/s13287-022-02941-w.

## Introduction

Type-1 diabetes (T1D) is a metabolic disease characterized by the autoimmune destruction of insulin-secreting pancreatic beta (*β*) cells. Hyperglycemia is a common problem of diabetics due to a lack of or low insulin secretion [1–7].

The International Diabetes Federation (IDF) introduces diabetes as one of the fastest-growing global health emergencies of this century and estimates that in 2019, 463 million people worldwide had diabetes. This number is expected to increase to 578 million by 2030 and 700 million by 2045 [8]. Among adults diagnosed with diabetes, about 5.2–5.6% have T1D [9, 10]. The American Diabetes Association (ADA) reported an increased number of affected children with T1D and an increase in prevalence in most sex, age, and ethnic subgroups, in addition to an increased incidence in almost all age groups [5].

In 2019, the IDF reported that 7808 children and adolescents between zero and 19 years old had T1D in Iran [8]. The innate and adaptive immune systems are deregulated in T1D, resulting in pancreatic islet *β*-cell destruction through CD4 (cluster of differentiation 4) and CD8 T-lymphocytes, natural killer (NK) cells, and B-lymphocyte infiltration following the invasion of dendritic cells and macrophages [11–13]. Immune cells and their secretory factors, such as pro-inflammatory cytokines, make an inflammatory environment that gradually mediates *β*-cell death. As a result, patients’ physiological blood glucose regulation fails, and diabetes complications endanger their life expectancy and quality of life (QOL) [11, 13, 14].

Diabetes causes severe chronic complications with irreversible multi-organ damage in patients. These complications include diabetic nephropathy, neuropathy, retinopathy, and cardiovascular diseases [1, 15–17].

T1D Patients’ survival depends on daily doses of exogenous insulin (EI) to balance their blood glucose levels. Although this is the standard treatment, it does not provide an adequate physiological response and cannot prevent the progressive degeneration of islet *β*-cells; consequently, these patients might experience hypoglycemic episodes [1, 2, 18–20].

Current therapies for T1D have significant limitations. Insulin injections are the first-line treatment with different challenges: long-term dependency, difficulty with EI level management, and potential insulin resistance with long-term usage. Second-line treatment, pancreas transplantation has faced multiple obstacles such as donor shortages, the possibility of transplant rejection, usage of immunosuppressive drugs, and high risks with an organ transplantation surgery [4, 21]. Thus, improved diabetes treatment methods that can address these limitations will be applicable in diabetes research and future management [21].

Cell-based therapy could ameliorate diabetes complications by providing better glycemic control [22]. Different cell therapy strategies for T1D include cell replacement, cell regeneration, and cell reprogramming. Various stem cells that have been tested for cellular therapy include hematopoietic stem cells (HSCs), induced pluripotent stem cells (iPSCs), and mesenchymal stem cells (MSCs) [2, 23, 24]. Other potential cell-based treatments for T1D include islet transplantation, transplantation of encapsulation devices, polyclonal regulatory T (T reg) cell transplantation, and alterations to the immune system response [19, 25–29].

To date, MSCs are the most studied cell population in clinical trials. The therapeutic capacity of MSCs is based on their ease of isolation, ability to differentiate into multiple cell types, low immunogenicity, abundant source, minimal ethical concerns, and, most notably, their release of biological factors that can alleviate impaired tissues [30]. The findings of a preclinical study showed that MSCs could be effective as a distant immunomodulator in addition to their homing to injured sites [31].

MSCs have a mesodermal origin and are defined as multipotent cells that can adhere to plastic; self-renew; express specific surface antigen markers (CD73, CD90, CD105); lack hematopoietic antigen expressions (CD45, CD34, CD14 or CD11b, CD79*α* or CD19, HLA-DR (Human Leukocyte Antigen—DR isotype)); and can differentiate into osteoblasts, adipocytes, and chondroblasts [30, 32]. MSCs are derived from many sources such as the bone marrow (BM), umbilical cord, umbilical cord blood (UCB), adipose tissue, and dental pulp [33–35].

Immunomodulatory therapeutic approaches can preserve residual *β*-cells in recent-onset T1D patients with a greater amount of functional *β*-cell mass [11, 36].

MSCs have a superior biosafety profile and negligible risk of tumorigenicity compared with induced pluripotent or embryonic stem cells [37]. Researchers have proposed using MSCs as an attractive therapy to ameliorate or reverse diabetes because of their systemic immunomodulatory and regenerative properties, no immunogenicity due to a lack of major histocompatibility complex (MHC) class II, and ability to home to damaged pancreatic islets and local lymph nodes [11, 13, 38–45].

We considered the promising features of MSCs and designed a randomized placebo-controlled phase I/II clinical trial. In this trial, we sought to examine the safety and efficacy of autologous bone marrow-derived MSCs as a treatment for newly diagnosed patients with T1D.

## Materials and methods

### Materials

Materials used in this study are listed in Additional file 1: Table S1.

## Methods

### Study design

A triple-blinded parallel randomized placebo-controlled trial was conducted between July 2015 and January 2020 at Royan Institute, Tehran, Iran, in collaboration with the Growth and Development Research Center of Tehran University of Medical Sciences, Tehran, Iran. The Research Ethics Committee at Royan Institute approved this study (approval code: IR.ACECR.ROYAN.REC.1394.50), and the study was conducted under the Declaration of Helsinki. The trial was registered at the Iranian Registry of Clinical Trials (IRCT) with the identifier IRCT ID: IRCT2016070428786N1 and at the U.S. National Institutes of Health (ClinicalTrials.gov) with the related identifier NCT04078308. Of note, we originally designed this study as a crossover study; however, our data analysis showed that the residual effects of the MSC transplantation were present after the 12-month follow-up (Additional file 1: Table S2). Therefore, the MSC versus placebo comparison was performed during the initial 12-month follow-up, and early versus late transplantation was compared in the second-year follow-up.

### Study population

**Fig. 1:**
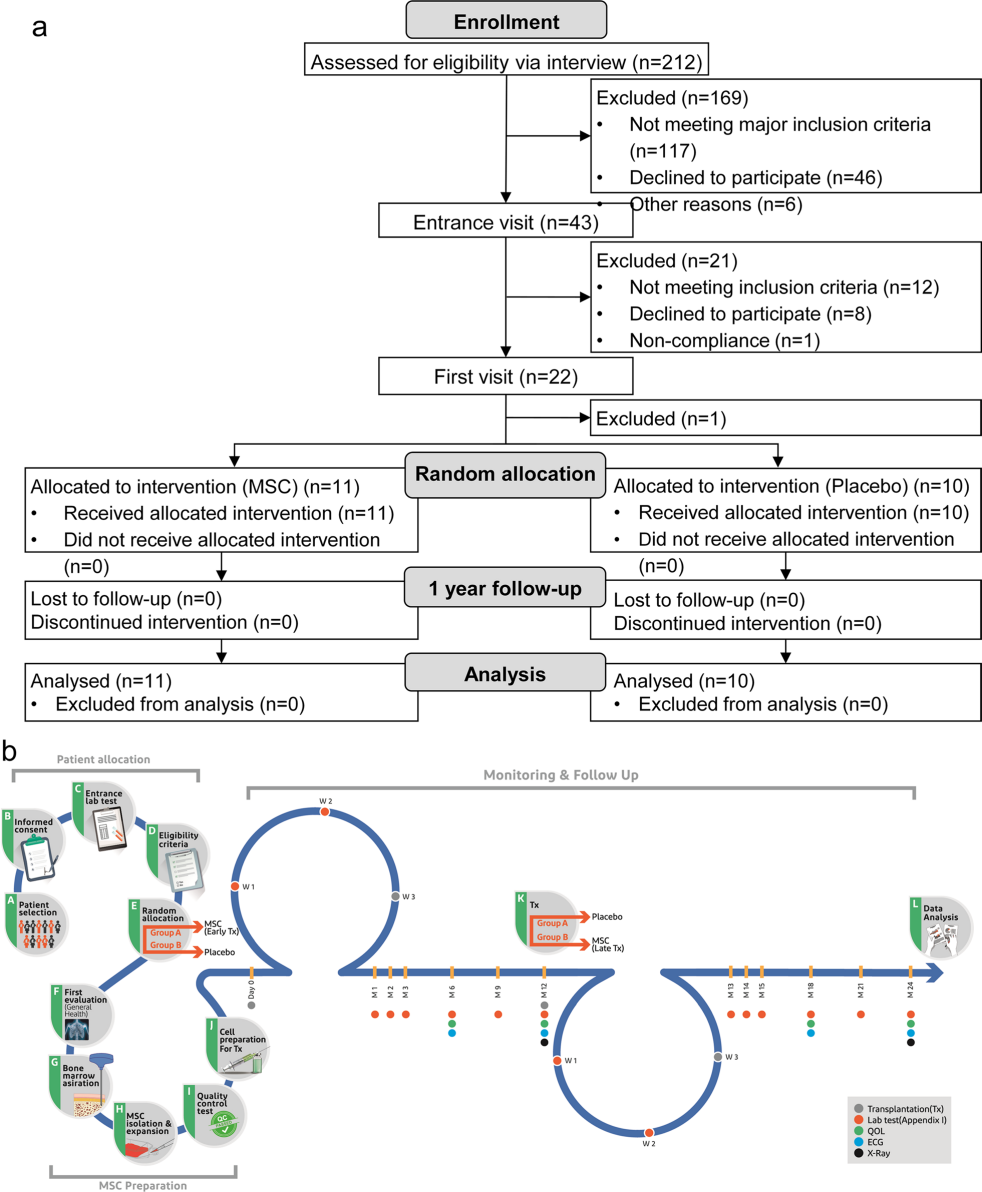
**a** CONSORT flow diagram. A total of 212 patients were assessed for eligibility, of which 43 were selected for further verification of the eligibility criteria. Finally, 21 participants were selected and randomly assigned to either the mesenchymal stem cell (MSC) or placebo group. The participants were followed for one year and then analyzed for study outcomes. **b** Schematic description of the study. The process of this clinical trial is generally divided into three main steps, including Patient allocation, MSC preparation, and Monitoring and Follow up. Briefly, selected patients gave informed consent, and after checking full eligibility criteria, they were randomly allocated to either receive MSC or Placebo. Then patients who underwent the first evaluation were subjected to bone marrow aspiration. MSCs were isolated from harvested bone marrow and expanded via culturing in a cleanroom facility. The cells that passed quality-control tests were intravenously injected into group A (early transplantation), while patients in group B received normal saline as a placebo. All patients were followed up for one year, and then the patients in group A received a placebo, whereas patients in group B received MSC (late transplantation). Data were analyzed after 12 and 24 months of follow-up

A total of 21 patients (age: 8 to 40 years) enrolled in this study. The patients were diagnosed with T1D according to diagnostic criteria and the ADA guidelines [5] within six weeks before enrollment based on previous studies [46, 47] and were already under classic insulin therapy. Patients were enrolled between July 2015 and January 2018 if they met the following inclusion criteria: evaluated fasting C-peptide level ≥ 0.3 nmol/L and presence of at least one of three autoantibodies against pancreatic *β* cells (islet cell antibody [ICA], glutamic acid decarboxylase antibody [GADA], or insulinoma associated-2 antibody [IA-2A]). The exclusion criteria consisted of pregnancy or breastfeeding; cancer; any acute or severe diseases (cardiac, pulmonary, hepatic, kidney, mental, or other diseases); positive test results for human immunodeficiency virus (HIV), human T-lymphotropic virus (HTLV), hepatitis B virus (HBV), hepatitis C virus (HCV), or cytomegalovirus (CMV); other immune-deficiencies; hyperesthesia; or history of severe ketoacidosis. Figure 1a presents the CONSORT (Consolidated Standards of Reporting Trials) flow diagram of the study population and their respective group assignment. All participants, their parents or legal guardians (when required for pediatric participants) received comprehensive oral and written information about the nature and possible consequences of the study. They signed a written informed consent form before participating in this clinical trial.

### Randomization and masking

Patients were randomly assigned to either the MSC or the placebo group by block randomization. A block size of four was used for randomization, and the sequences were calculated by Random allocation software v. 2.0. The research coordinator had exclusive access to the numbered, sealed, opaque envelopes that indicated each patient’s randomized treatment assignment.

### Blinding

In this triple-blinded study, the participants, care providers, and outcomes assessors were blinded to the treatment assignments. To protect the blinding, two different physicians conducted the intervention, patient assessments, and follow-ups. The vials that contained either the MSCs or the placebo were similar. Only the specific randomization code assigned to an individual patient was visible on the vial. The person who prepared the cells and the research coordinator were aware of the vials’ exact contents.

### Study interventions

Participants were randomly assigned to receive either two doses of 1 × 10^6^ autologous MSCs per kilogram of the patient’s body weight or placebo at weeks 0 and 3. Besides, we compared the effects of early versus late transplantation of MSCs. Newly diagnosed T1D patients who received the MSCs transplantation during the first year were considered the early transplantation group (Early Tx). The late transplantation group (Late Tx) were patients who received the placebo during the first year of the study and were then assigned to receive the MSCs transplantation at least one year after their diabetes diagnosis. According to the eligibility criteria, all patients underwent baseline laboratory blood tests, EKG (electrocardiogram), and chest X-ray assessments to confirm their general health. After randomization, bone marrow was aspirated from all the participants’ iliac crests while under general anesthesia.

### Mesenchymal stem cell (MSC) preparation and transplantation

Clinical-grade MSCs were isolated from bone marrow, expanded in passages 2–3, and cryopreserved under good manufacturing practice (GMP) conditions at Royan Institute, as previously described [48]. The cells were maintained in culture up to 24 h post thawing to recover from the cryopreservation prior to transplantation. The release criteria for the therapeutic use of isolated MSCs included the absence of viral, microbial, fungal, and mycoplasma contamination; a less than acceptable level of endotoxin; normal karyotype; and cell viability of more than 95% (Additional file 1: Fig. S1–S3, S4a). MSCs passed the minimal criteria proposed by the International Society for Cellular Therapy (ISCT) for MSC definition. The MSCs were assessed by flow cytometry for their ability to express CD105, CD90, CD44, CD73, and lack of CD34 and CD45 expression (Additional file 1: Fig. S4). Also, MSCs had normal morphology and were differentiated into osteocytes, adipocytes, and chondrocytes to confirm their identity (Additional file 1: Fig. S5). Differentiation assay was not performed on all patient samples. However, it was done at the protocol set up steps and intermittently for process validation.

Three to four weeks after bone marrow aspiration, 1 × 10^6^ autologous MSCs per kilogram of the patient’s body weight were injected for each dose of two infusions at weeks 0 and 3. Cells were suspended in 100 ml normal saline (0.9%) and administrated intravenously (IV) within ~ 30–40 min. The placebo group received a similar amount of normal saline. The first transplantation was regarded as day zero in this study. The cell dosage and route of the transplantation were selected based on the previous studies and amassed experience of in-house physicians for using MSCs for their therapeutic potential in different diseases for over ten years, in which 2 million cells per kg of patients’ body weight is the most common dosage of MSCs in the IV route [11, 49].

### Patient follow-up

The patients were followed for one year after the first infusion to assess safety and efficacy parameters. During this time, the patients were seen at weeks 1, 2, and 4, and months 2, 3, 6, 9, and 12 for follow-ups (Additional file 1: Table S3). Furthermore, the patients recorded blood glucose self-monitoring (at least two or three times per day) during this period to evaluate treatment safety and efficacy.

### Study outcomes

#### Primary outcome: Safety

The patients provided medical histories and underwent physical examinations at each outpatient visit to monitor for treatment-related adverse events and hypoglycemic episodes. Safety was assessed based on the Common Terminology Criteria for Adverse Events version 5 (CTCAE v.5). The number of hypoglycemic episodes was assessed by evaluating patients’ blood glucose monitoring sheets. Hypoglycemia was defined as each blood glucose recording below 70 mg/dl according to CTCAE v.5.

#### Secondary outcome: Efficacy

Efficacy was defined as changes from baseline in fasting blood sugar (FBS), two-hour postprandial glucose test (2hpp), C-peptide, serum levels of Glycated hemoglobin (HbA1c), daily dose of injected EI, and lability index (LI).

FBS and 2hpp were evaluated using GLUC3 kit, C-peptide using Elecsys C-peptide kit, and HbA1c using Tina-quant® HbA1c Gen. 3. These parameters were measured by COBAS® 6000 analyzer and COBAS INTEGRA® 400 plus analyzer Roche Diagnostics.

Serum levels of interleukin 6 (IL-6), tumor necrosis factor-alpha (TNF-*α*), interleukin 4 (IL-4), interleukin 10 (IL-10), and transforming growth factor-beta 1 (TGF-*β*1) were evaluated using Enzyme-linked immunosorbent assay (ELISA) utilizing the following kits: Human IL-6 ELISA Kit, Human TNF-*α* ELISA Kit, Human IL-4 Quantikine ELISA Kit, Human IL-10 Quantikine ELISA Kit, and Human TGF- *β*1 Quantikine ELISA Kit.

Patients’ QOL was assessed by the 36-item Short Form Survey (SF-36) and Diabetes QOL (DQOL) questionnaires. The effects of physical exercise on metabolic and immunologic indices between the MSC transplantation and placebo groups were compared in some patients.

The percentages of CD4 + CD25 + Foxp3 + T reg cells were also assessed in patients’ peripheral blood before the MSCs transplantation and 48 h after transplantation, as described before [50]. Briefly, a Ficoll density gradient was used to separate peripheral blood mononuclear cells (PBMCs) from collected blood in ethylenediaminetetraacetic acid (EDTA)-containing tubes. The PBMCs were incubated with FITC conjugated anti-CD4 and PE-conjugated anti-CD25 antibodies to stain the surface markers. Then, the PBMCs were incubated with fixation and permeabilization buffer BD Cytofix/Cytoperm™, washed with BD Perm/Wash buffer, and stained with PerCP anti-Foxp3 antibody. The stained PBMCs were characterized by a flow cytometer (BD FACSCalibur™, BD Biosciences, San Jose, CA, USA) equipped with BD CellQuest Pro software. The acquired data were analyzed by FlowJo software (Version: 7.6.1, BD Biosciences). Isotype controls were utilized to assist in precise compensation and evaluate antibody specificity.

### Sample size

The calculated sample size was based on HbA1c improvement over 12 months in the MSC group versus the placebo group. We assumed the anticipated effect size of *f* = 0.52, type I error of 0.05, test power of 80%, and correlation coefficient of 0.5 for repeated measurements based on Hu et al.’s study [11]. A total sample size of *n* = 20 was calculated with G*Power 3.1 (University of Kiel, Germany) using repeated measures analysis of variances (ANOVA).

### Statistical analysis

All data were collected and stored at Royan Institute. Primary and secondary outcome analyses were performed on all randomized, eligible patients. All analyses were based on a statistical analysis plan prepared by a statistician blinded to the study assignments, and all analytic conclusions were based on masked data reviews.

We used the modified intention to treatment (MITT) method for the standard primary analysis. Patients were analyzed according to their randomized arm. Missing data were excluded at each time point, and the analysis was based on the available cases at each time point.

Analyses were performed using Stata software (StataCorp. 2015. *Stata Statistical Software: Release 14*. College Station, TX: StataCorp LP.), and the graphs were generated with GraphPad Prism version 9.1.1 for Windows, GraphPad Software, San Diego, California, USA, www.graphpad.com. The significance level for all analyses was set at *α* = 0.05. Baseline continuous characteristics are reported as mean ± standard deviation, and categorical variables are reported as *N*.

Interventional effects on primary and secondary outcomes were evaluated using a generalized estimating equation (GEE) for longitudinal data with an identity link and an exchangeable correlation matrix to give population-averaged estimates. The placebo group was considered the reference group to evaluate the effects of the intervention. The Early Tx group was used as the reference group to evaluate the effects of new-onset on intervention. Also, we assessed the residual data from the GEE model. GEE models were used to evaluate changes from baseline to 3, 6, 9, and 12 months for primary and secondary continuous outcomes measured longitudinally. Groups were included in the models as the covariate, and visit time was the categorical measure. The interaction between groups and time was considered in the model, and confidence intervals (CI) and *P*-values were adjusted by the confounders of body mass index (BMI), age, and sex.

## Results

In the current study, data are presented stepwise, starting from safety to efficacy results. The efficacy results are summarized in 3 main groups: metabolic, immunologic, and QOL indices. This pattern is followed for both MSCs vs. Placebo and Early vs. Late transplantation. The rest of the results are provided at the end of the results section. The charts are provided in the main text to facilitate data interpretation, and the corresponding tables are available in the supplementary data for experts demanding detailed information on data analysis.

### Study population

A total of 212 patients were screened for eligibility from July 2015 to January 2018, in which 21 met the eligibility criteria and were randomly assigned to either the MSC transplantation or the placebo group (Fig. 1a). The patients were followed up for a year after receiving their first injection. Figure 1b provides a brief description of the trial steps and timeline. The baseline demographic and clinical characteristics were similar in both groups (Table 1).

**Table 1:** Baseline demographic characteristics of the study participants (*n* = 21)

General characteristics	Placebo^a^ (*n* = 10)	MSC^a^ (*n* = 11)
*Demographics*		
Age (years)	11.50 ± 2.63	10.27 ± 1.67
BMI (kg/m^2^)	18.91 ± 3.41	16.75 ± 2.57
Male/Female	5/5	6/5
*Residence*		
Local (Tehran Province)/other	3/7	6/5
*Auto-antibodies (AA) (negative/positive)*		
ICA	6/4	6/5
GADA	0/10	3/8
IA-2A^*^	5/3	3/7
*Metabolic indices*		
FBS (mg/dl)	149.4 ± 51.97	165.27 ± 97.49
2hpp (mg/dl)	234.7 ± 80.39	269.00 ± 173.29
HbA1c (%)	7.85 ± 1.45	8.63 ± 2.19
C-peptide (ng/ml)	0.92 ± 0.57	0.72 ± 0.38
EI (IU/kg/day)	0.71 ± 0.30	0.78 ± 0.44
LI (mmol/l2/h.week-1)	42.09 ± 44.31	29.44 ± 35.50
*Cytokines*		
Pro-inflammatory		
IL-6 (ng/l)	86.66 ± 30.11	106.96 ± 57.67
TNF-*α* (pg/ml)	68.29 ± 34.49	94.30 ± 55.55
*Anti-inflammatory*		
IL-4 (pg/ml)	325.02 ± 179.45	249.84 ± 208.04
IL-10 (pg/ml)	412.38 ± 336.84	236.22 ± 221.86
TGF-*β*1 (pg/ml)	802.58 ± 386.44	539.96 ± 529.99
*Quality of life questionnaires*		
DQOL	75.97 ± 11.16	75.91 ± 9.69
SF-36	77.09 ± 13.46	74.43 ± 17.98

BMI, Body mass index; ICA, Islet cell antibody; GADA, Glutamic acid decarboxylase antibody; IA-2A, Insulinoma associated-2 antibody; FBS, Fasting blood sugar; 2hpp, Two-hour postprandial; HbA1c, Glycated haemoglobin; MSC, Mesenchymal stem cell; EI, Exogenous insulin; LI, Lability index; IL-6, Interleukin 6; TNF-*α*, Tumor necrosis factor-alpha; IL-4, Interleukin 4; IL-10, Interleukin 10; TGF-*β*1, Transforming growth factor-beta 1; DQOL, Diabetes Quality of Life; SF-36, 36-Item Short Form Survey; QOL, Quality of life

*Other missing

^a^Variables are expressed as mean ± standard deviation and count

### Major complications and death

We did not find any major complications or deaths among participants in this study.

### Losses and exclusions

Two patients, who received the allocated treatment in the first year and completed the 12 months of follow-up, declined to participate in the second course of treatment due to personal issues unrelated to treatment.

### Primary outcomes: Safety

#### Transplantation-associated adverse events

There were no major transplantation-related adverse events observed in either group. One adverse event potentially unrelated to the MSC transplantation was a mild injection site reaction observed in two patients (one in each group). A grade 3 urticaria was observed in P-1 during the first injection. Symptoms were relieved with the administration of a mild anti-inflammatory medication. A mild increase in lymphocytes occurred in P-2 and mild hyperkalemia in P-4 from the MSC group, both of which occurred 12 months after transplantation and appeared unrelated to the treatment procedure.

Other adverse events reported in the MSC and placebo groups were negligible or reported only in the placebo group, mainly without any medical intervention.

Table 2 lists the observed adverse events in this trial. The observed adverse events were not serious, and, when necessary, the required medical interventions were applied.

**Table 2:** List of observed adverse events. Observed adverse events were not serious, and the required medical considerations were applied in case they needed

No	MedDRA code	CTCAE term	Observed grade	Placebo (*n* = 10)		MSC (*n* = 11)	
				*N*	Patient ID/time	*N*	Patient ID/time
*Investigations*							
1	10001551	Alanine aminotransferase (ALT) increased	1	1	P-11/M6		
2	10001675	Alkaline phosphatase (ALP) increased	1	4	P-3/M6 P-8/M6, 12 P-9/M12	5	P-2/M6, 9, 12 P-6/M6 P-10/M6
3	10003481	Aspartate aminotransferase (AST) increased	1	2	P-9/M12 P-11/M6		
4	10025258	Lymphocyte count increased	2			1	P-2/M12
5	10047900	Weight loss	1	1	P-18/M6		
*General disorders and administration site conditions*							
6	10016558	Fever	1	1	P-18/M1		
7	10022095	Injection site reaction	1	1	P-3/M1	1	P-6/W1
			2	1	P-5/M1	1	P-6/M1
8	10033371	Pain (epigastric region)	1	1	P-12/M3	1	P-13/M2
*Metabolism and nutrition disorders*							
9	10020587	Hypercalcemia	1	1	P-7/M12		
10	10020647	Hyperkalemia	4			1	P-4/M12
11	10020907	Hyperuricemia	1	1	P-18/M12		
12	10020949	Hypocalcemia	2	1	P-12/M12		
14	10021018	Hypokalemia	1	1	P-14/M6		
15	10021038	Hyponatremia	1	2	P-3/M12 P-9/M12		
*Skin and subcutaneous tissue disorder*							
16	10046735	Urticaria	3			1	P-1/First Injection
*Endocrine*							
17	10021114	Hypothyroidism	1	1	P-8/M9		
*Neurology*							
18	10037175	Psychiatric disorders	1	1	P-8/M6		

MSC, Mesenchymal stem cell; M, Month; W, Week; N, Number of events

A complete list of the assessed adverse events is found in Additional file 1: Table S4.

#### Hypoglycemia

**Fig. 2:**
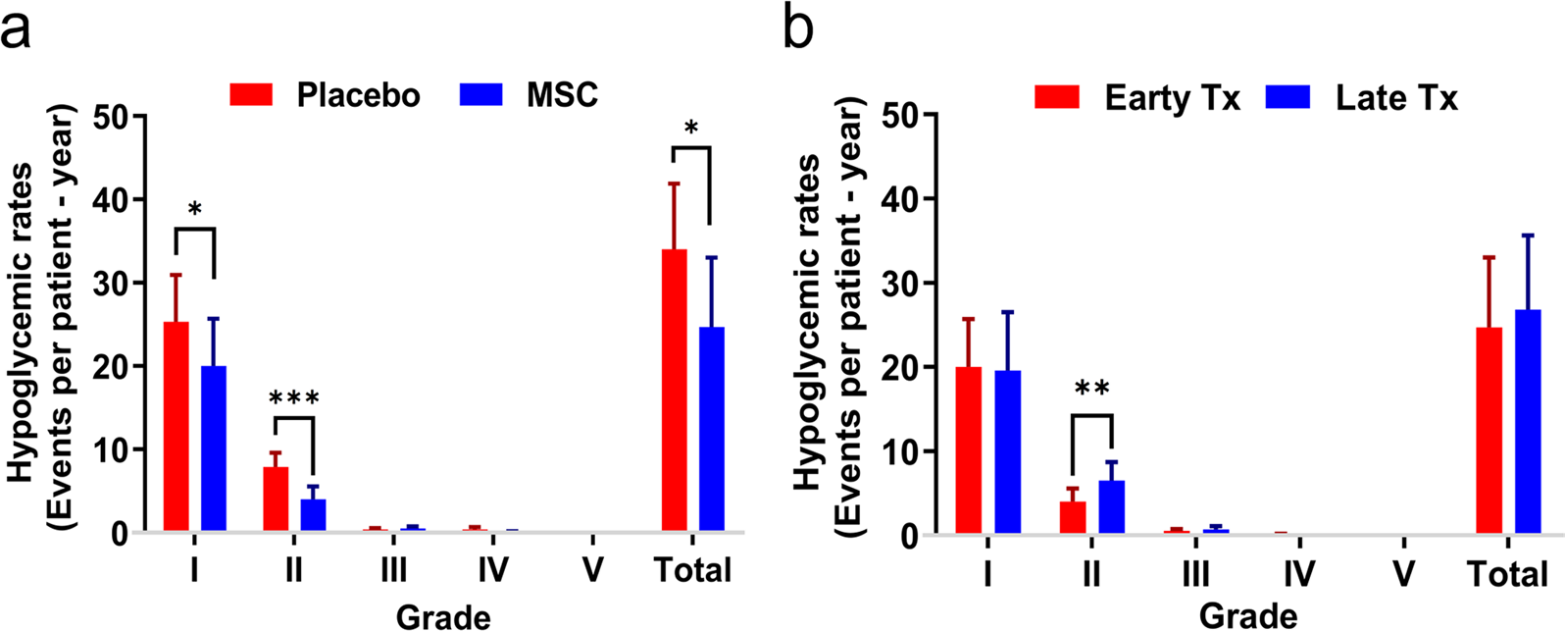
Hypoglycemic rates (events per patient—year) **a** Mesenchymal stem cell (MSC) transplantation versus placebo, **b** Early versus late transplantation (Grade I: < lower limit of normal (LLN)—55 mg/dL; Grade II: < 55–40 mg/dL; Grade III: < 40–30 mg/dL; Grade IV: < 30 mg/dL, life-threatening consequences, seizures; Grade V: Death. Error bars represent the standard deviation. *P < 0.05, **P < 0.01, ***P < 0.001)

Hypoglycemic events are defined as observing blood glucose levels lower than 70 mg/dl. The number of hypoglycemic events was considered not only a safety parameter but also an efficacy outcome. These events are graded from I to V according to the severity in the CTCAE v.5, as follows: grade I (< lower limit of normal [LLN]—55 mg/dL); grade II (< 55–40 mg/dL); grade III: (< 40–30 mg/dL); grade IV (< 30 mg/dL, life-threatening consequences, seizures); and grade V (Death). The MSC transplantation group had significantly lower numbers of grade I (*P* = 0.0457) and II (*P* < 0.001) hypoglycemic episodes and a total number of hypoglycemic events (*P* = 0.0164) compared to the placebo group (Fig. 2a). Early transplantation of MSCs significantly reduced the number of grade II hypoglycemic events compared to late transplantation of MSCs (*P* = 0.008, Fig. 2b).

### Secondary outcome: Efficacy

#### Mesenchymal stem cells (MSCs) versus placebo

##### Metabolic indices

**Fig. 3:**
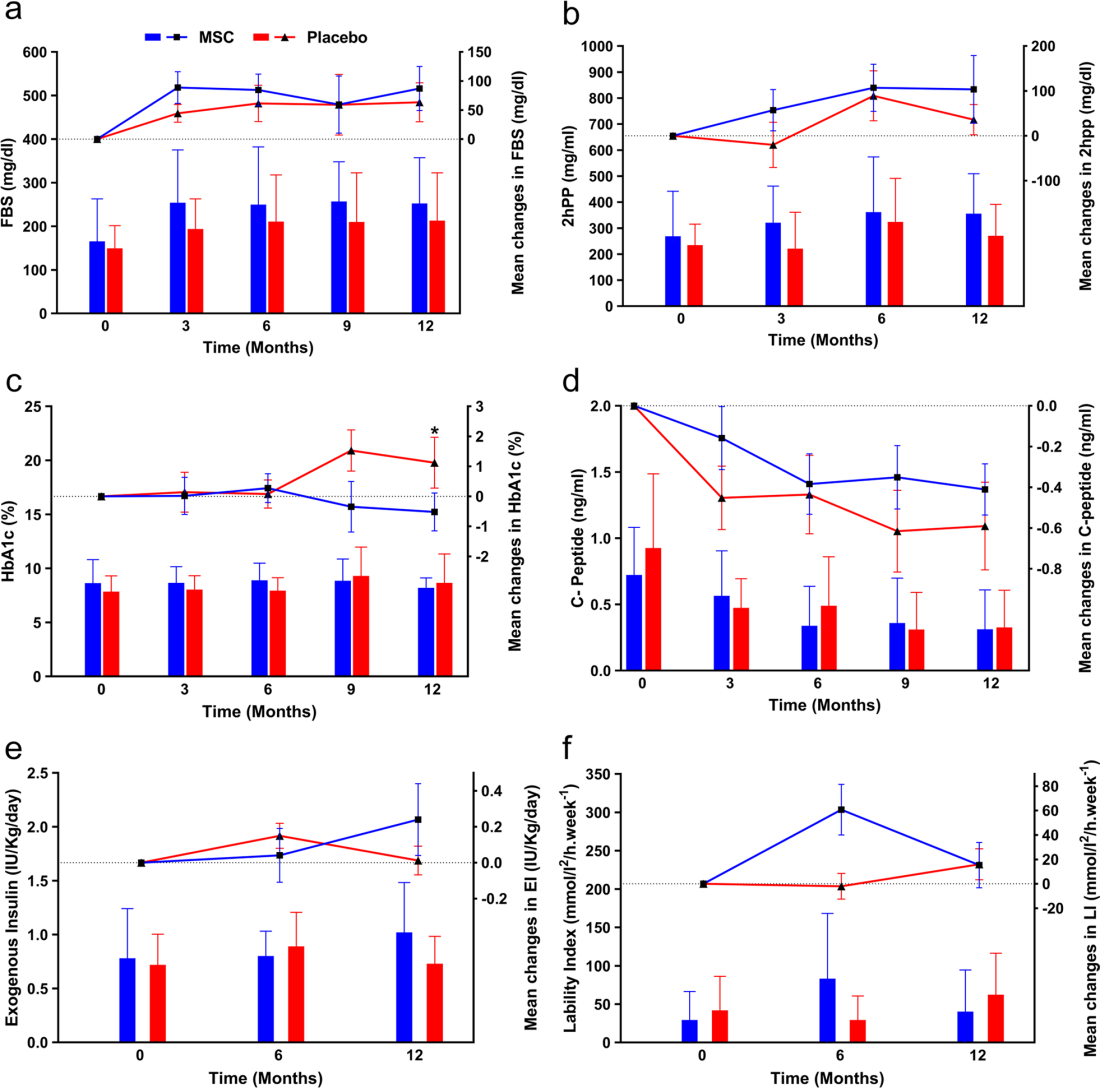
Comparison of metabolic indices between the mesenchymal stem cell (MSC) and placebo groups. The left Y-axis represents the mean in each group drawn by column charts, and the right Y-axis belongs to the mean differences compared to the baseline drawn by scatter plots. **a** Fasting blood sugar (FBS), **b** Two-hour postprandial (2hpp), **c.** Glycated hemoglobin (HbA1c), **d** C-peptide, **e** Exogenous insulin (EI), **f** Lability index (LI). Error bars represent the standard deviation for the bar charts and the standard error of the mean for scatter plots. Chart legend for the entire picture is shown in part (**a**). *P < 0.05

There were no significant differences in FBS and 2hpp between the MSC and placebo groups (Additional file 1: Table S5, Fig. 3a, b). As shown in Additional file 1: Table S5, MSCs significantly reduced the percent of HbA1c 12 months after transplantation (*P* = 0.043). There were considerable mean changes in HbA1c at month 9 (*P* = 0.061, Fig. 3c). The mean differences in C-peptide levels between the two groups showed higher but insignificant changes that favored MSC transplantation (Fig. 3d). No significant changes in the amount of EI and LI were reported (Fig. 3e, f). However, although the 6-month follow-up showed a reduction in EI, there was a slight increase at the 12-month follow-up (Fig. 3e). The LI decreased at the 12-month follow-up in patients who received MSCs (Fig. 3f).

##### Immunologic indices

**Fig. 4:**
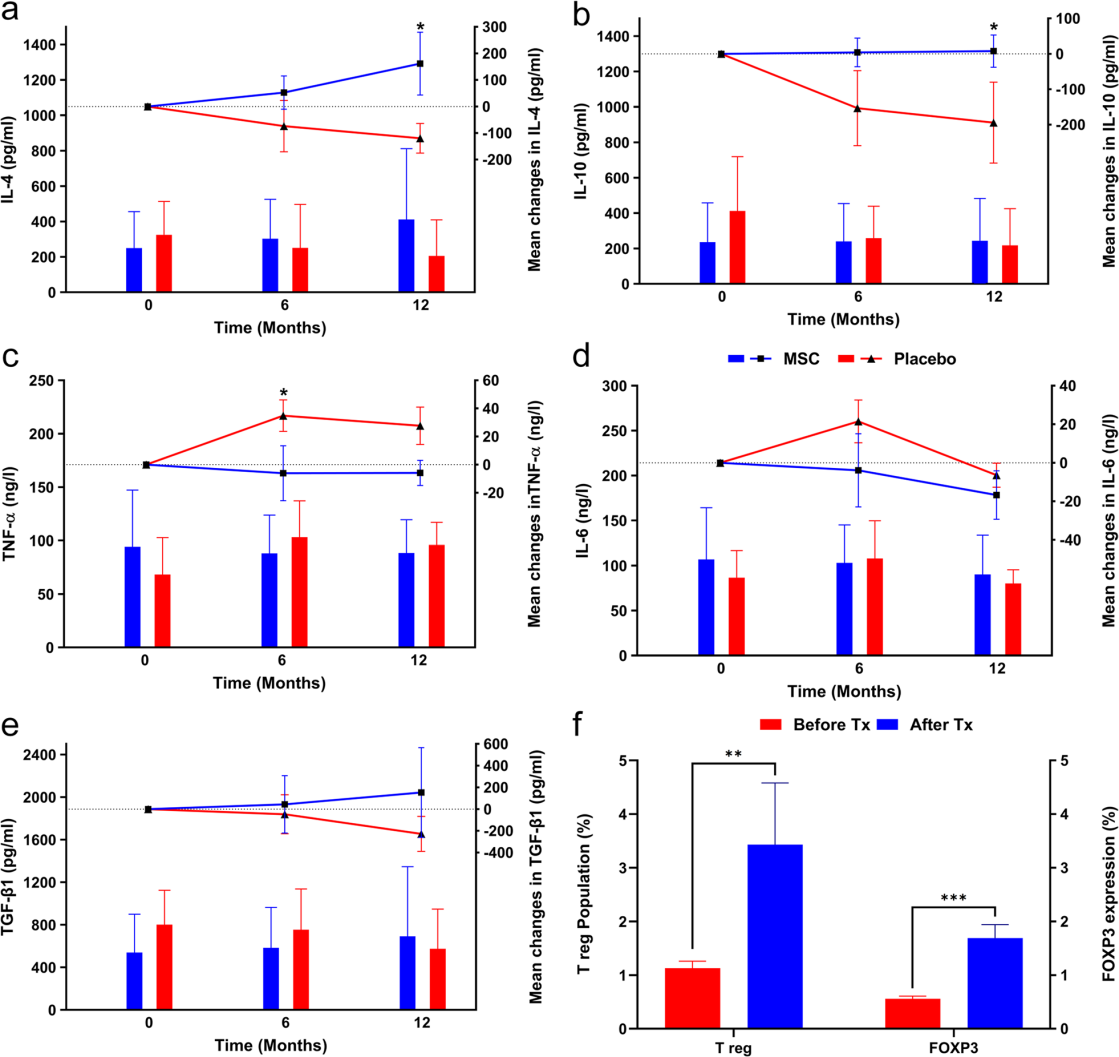
Comparison of cytokine levels between mesenchymal stem cell (MSC) and placebo groups. The left Y-axis represents the mean in each group drawn by column charts, and the right Y-axis belongs to the mean differences compared to the baseline drawn by scatter plots. **a** Interleukin 4 (IL-4), **b** Interleukin 10 (IL-10), **c** Tumor necrosis factor-alpha (TNF-*α*), Interleukin 4 (IL-4), **d** Interleukin 6 (IL-6), **e** Transforming growth factor-beta 1 (TGF-*β*1), **f** Regulatory T cell (T reg) population and Forkhead box P3 (FOXP3) expression before and 48 h after transplantation. Tx: Transplantation. Error bars represent the standard deviation for bar charts and the standard error of the mean for scatter plots. Chart legend for the entire picture is shown in part **a** unless otherwise indicated. *P < 0.05, **P < 0.01, ***P < 0.001

According to previous studies [42, 51], IL-6 and TNF-*α* have pro-inflammatory effects, whereas IL-4, IL-10, and TGF-*β*1 show anti-inflammatory effects. Transplantation of MSCs, as an immunomodulator, significantly increased the anti-inflammatory cytokines IL-4 (*P* = 0.032) and IL-10 (*P* = 0.30) (Fig. 4a, b; Additional file 1: Table S6). This effect persisted after 12 months (Fig. 4a, b; Additional file 1: Table S6). TNF-*α* levels significantly reduced after six months (*P* = 0.027) and showed a considerable decrease after 12 months (Fig. 4c, Additional file 1: Table S6). The changes in IL6 and TGF-*β*1 were not significant (Fig. 4d, e).

T reg cells are a group of immune cells that play an essential role in peripheral tolerance induction and maintenance; therefore, they modulate the immune response and prevent autoimmunity. Flow cytometry analysis of their antigens showed significant increases in T reg cell levels (*P* = 0.0088) and Forkhead box P3 (FOXP3) expression (*P* = 0.0004) 48 h after transplantation of the MSCs (Fig. 4f).

##### Quality of life (QOL)

DQOL questions are categorized into three subsections: satisfaction, impact, and worry. The SF-36 questions are categorized into eight subsections: physical functioning (PF), role—physical (RP), bodily pain (BP), general health (GH), vitality (VT), social functioning (SF), role—emotional (RE), and mental health (MH). Two main sections of the SF-36 consist of these eight subsections. Section A: Physical Health includes the PF, RP, BP, GH, and VT subsections, whereas section B: Mental Health includes the GH, VT, SF, RE, and MH subsections.

**Fig. 5:**
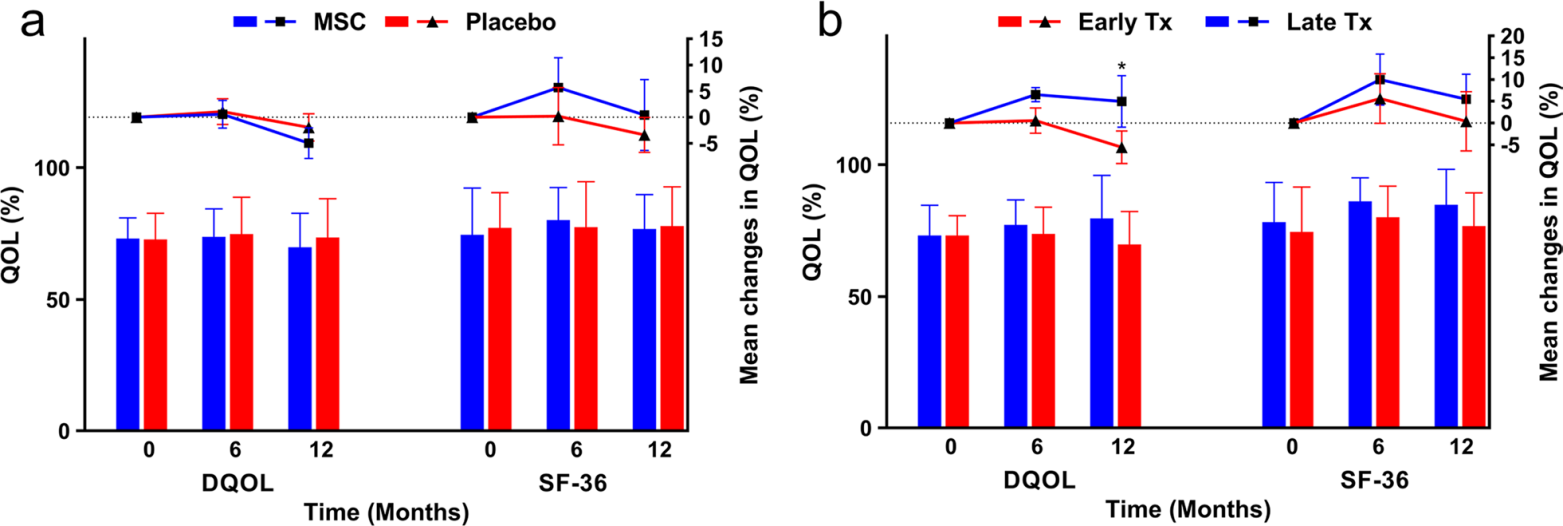
Quality of life (QOL) **a** Comparison of patients’ QOL between the mesenchymal stem cell (MSC) and placebo groups based on the Diabetes specific QOL (DQOL) and 36-Item Short Form Survey (SF-36) questionnaires. The left Y-axis represents the mean in each group drawn by column charts, and the right Y-axis belongs to the mean differences compared to the baseline drawn by scatter plots. There were no significant changes reported. Error bars represent the standard deviation for bar charts and the standard error of the mean for scatter plots. **b** Comparison of patients’ QOL between early (Early Tx) and late (Late Tx) transplantation of MSCs based on the DQOL and SF-36 questionnaires. The left Y-axis represents the mean in each group drawn by column charts, and the right Y-axis belongs to the mean differences compared to the baseline drawn by scatter plots. Error bars represent the standard deviation for bar charts and the standard error of the mean for scatter plots. *P < 0.05

After six months, patients reported a significant reduction in worry related to DQOL in the MSC group (*P* = 0.012). The MSC group reported a significant improvement in RP (*P* = 0.020) at the 6-month follow-up and a significant improvement in GH (*P* = 0.037) at the 12-month follow-up (Additional file 1: Table S7, Fig. 5a).

#### Early versus late transplantation

##### Quality of life (QOL)

Additional file 1: Table S8 shows that the total DQOL (*P* = 0.026) and social functioning subsection (*P* = 0.025) of the SF-36 questionnaire showed significant improvements in the Late Tx group at 12 months of follow-up (Fig. 5b).

##### Metabolic indices

**Fig. 6:**
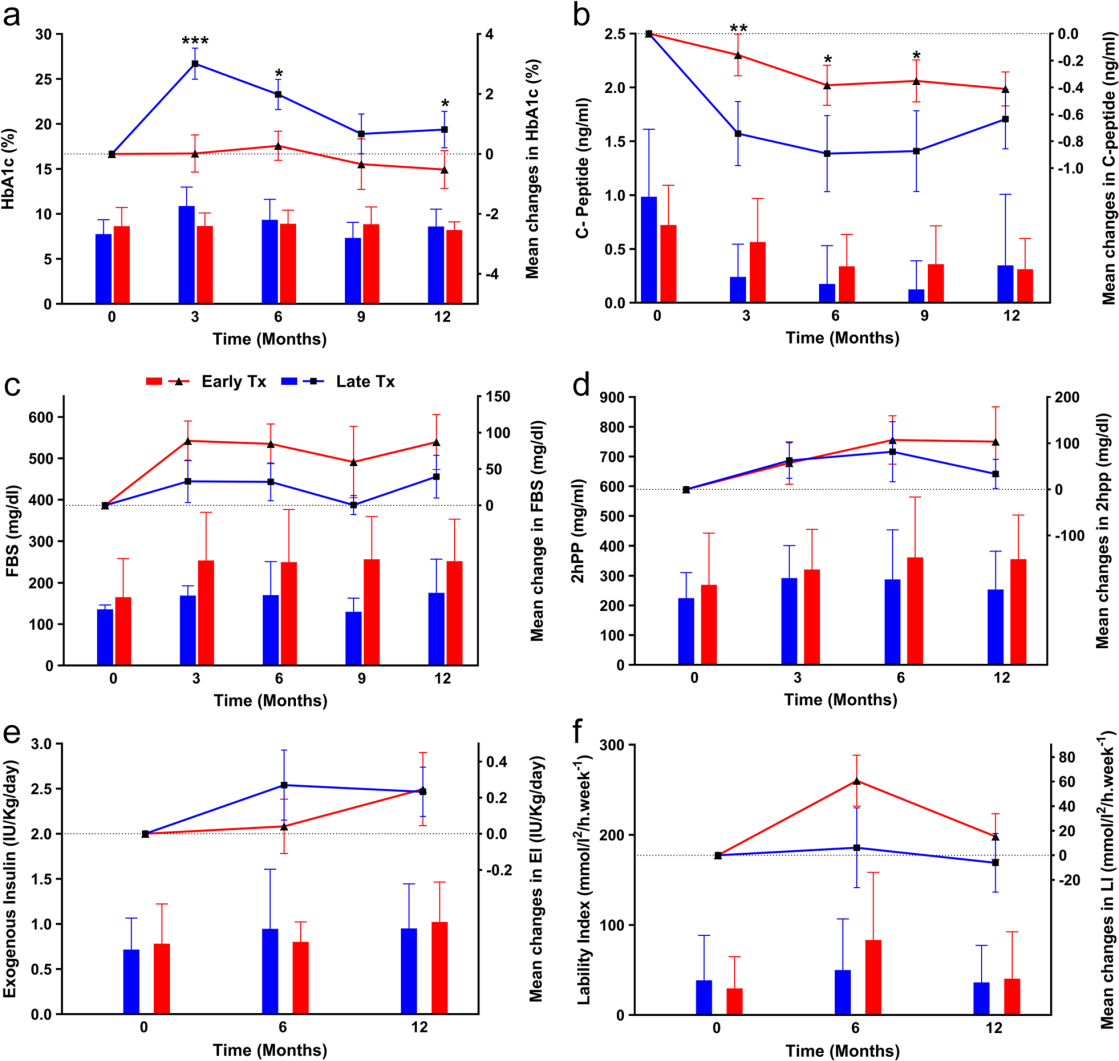
Comparison of metabolic indices between early transplantation (Early Tx) and late transplantation (Late Tx) groups. The left Y-axis represents the mean in each group drawn by column charts, and the right Y-axis belongs to the mean differences compared to the baseline drawn by scatter plots. **a** Glycated hemoglobin (HbA1c), **b** C-peptide, **c** Fasting blood sugar (FBS), **d** Two-hour postprandial (2hpp), **e** Exogenous insulin (EI), **f** Lability index (LI). Error bars represent the standard deviation for bar charts and the standard error of the mean for scatter plots. Chart legend for the entire picture is shown in part (**a**). *P < 0.05, **P < 0.01, ***P < 0.001

We compared the new-onset group that received MSC transplantation in the first year when they were newly diagnosed with T1D (Early Tx) and the patients who received the transplantation at least one year after their diagnosis (Late Tx). The results show that early transplantation of MSCs can significantly decrease the percentages of HbA1c at 3 (*P* < 0.001), 6 (*P* = 0.015), and 12 (*P* = 0.041) months after transplantation compared to the Late Tx group (Additional file 1: Table S9, Fig. 6a). Serum C-peptide levels in the Early Tx group were significantly higher than the Late Tx group at 3 (*P* = 0.003), 6 (*P* = 0.023), and 9 (*P* = 0.016) months of follow-up (Fig. 6b). However, the other evaluated indices were not significant (Additional file 1: Table S9, Fig. 6c–f).

##### Immunologic indices

**Fig. 7:**
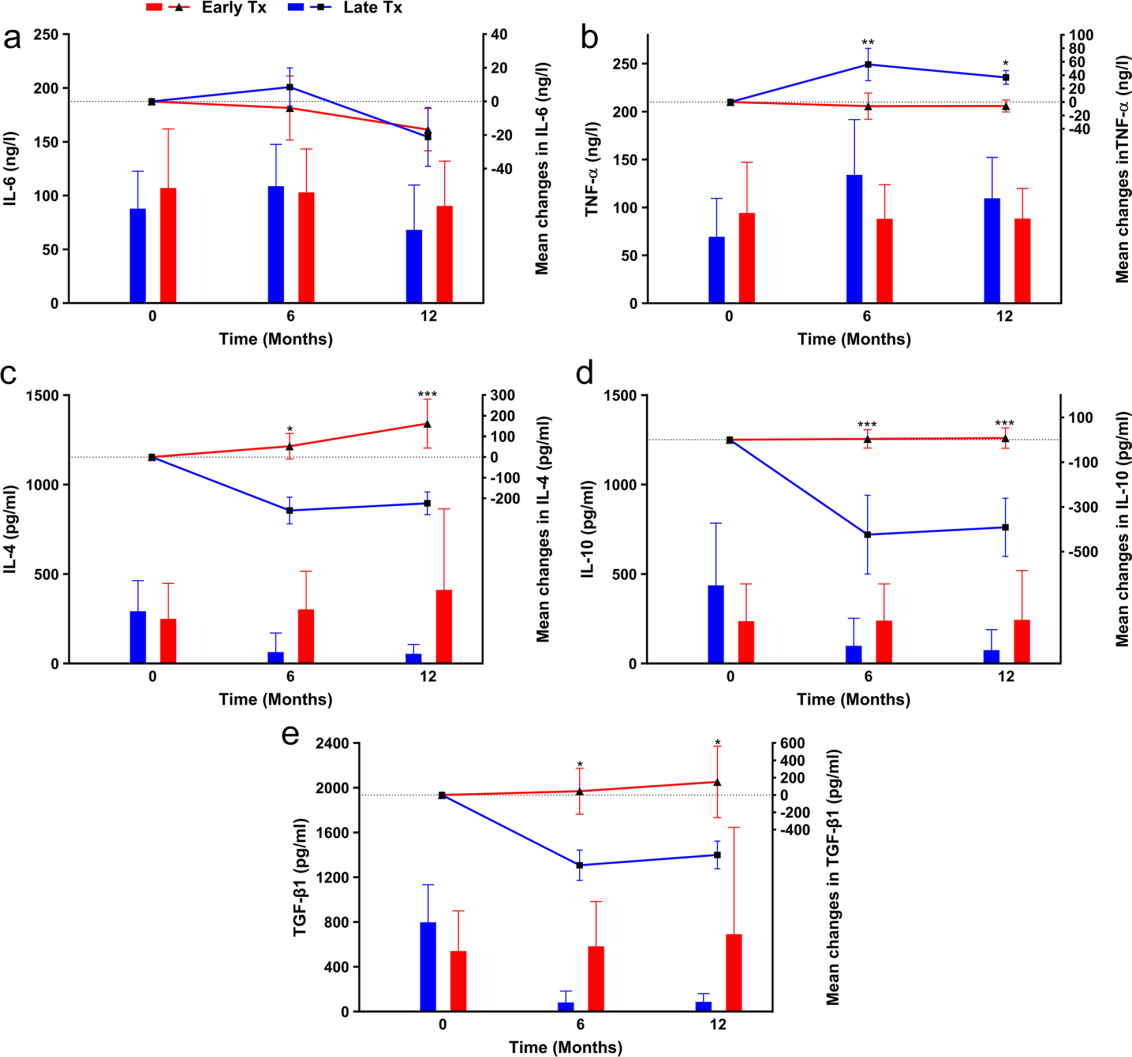
Comparison of cytokine levels between the early transplantation (Early Tx) and late transplantation (Late Tx) groups. The left Y-axis represents the mean in each group drawn by column charts, and the right Y-axis belongs to the mean differences compared to the baseline drawn by scatter plots. **a** Interleukin 6 (IL-6), **b** Tumor necrosis factor-alpha (TNF-*α*), **c** Interleukin 4 (IL-4), **d** Interleukin 10 (IL-10), **e** Transforming growth factor-beta 1 (TGF-*β*1). Error bars represent the standard deviation for bar charts and the standard error of the mean for scatter plots. Chart legend for the entire picture is shown in part (**a**). *P < 0.05, **P < 0.01, ***P < 0.001

TNF-*α* levels were significantly lower in the Early Tx group than the Late Tx group at the 6- (*P* = 0.002) and 12- (*P* = 0.028) month follow-ups. IL-6 levels were not significantly different between the groups (*P* = 0.877) (Additional file 1: Table S10, Fig. 7a, b). IL-4 and IL-10 levels were significantly higher in the Early Tx group compared to the Late Tx group at the 6- and 12-month follow-ups (P_6_ = 0.025, P_12_ = 0.001, and P_6_ < 0.001, P_12_ < 0.001, respectively) (Additional file 1: Table S10, Fig. 7c, d). TGF-*β*1 levels were significantly higher in the Early Tx group than the Late Tx group at the 6-month (*P* = 0.038) and 12-month (*P* = 0.016) follow-ups (Additional file 1: Table S10, Fig. 7e), which indicated a successful shift from pro-inflammatory to anti-inflammatory cytokines following MSC transplantation in newly diagnosed T1D patients.

#### Exercise

ADA guidelines recommend that children 5 to 18 years of age exercise at least three days each week, with more than 60 minutes of daily activity and 150 minutes of weekly exercise for adults [5, 52]. This study measured the metabolic and immunologic indices by considering the 3.5 h per week exercise cut-off for some patients.

**Fig. 8:**
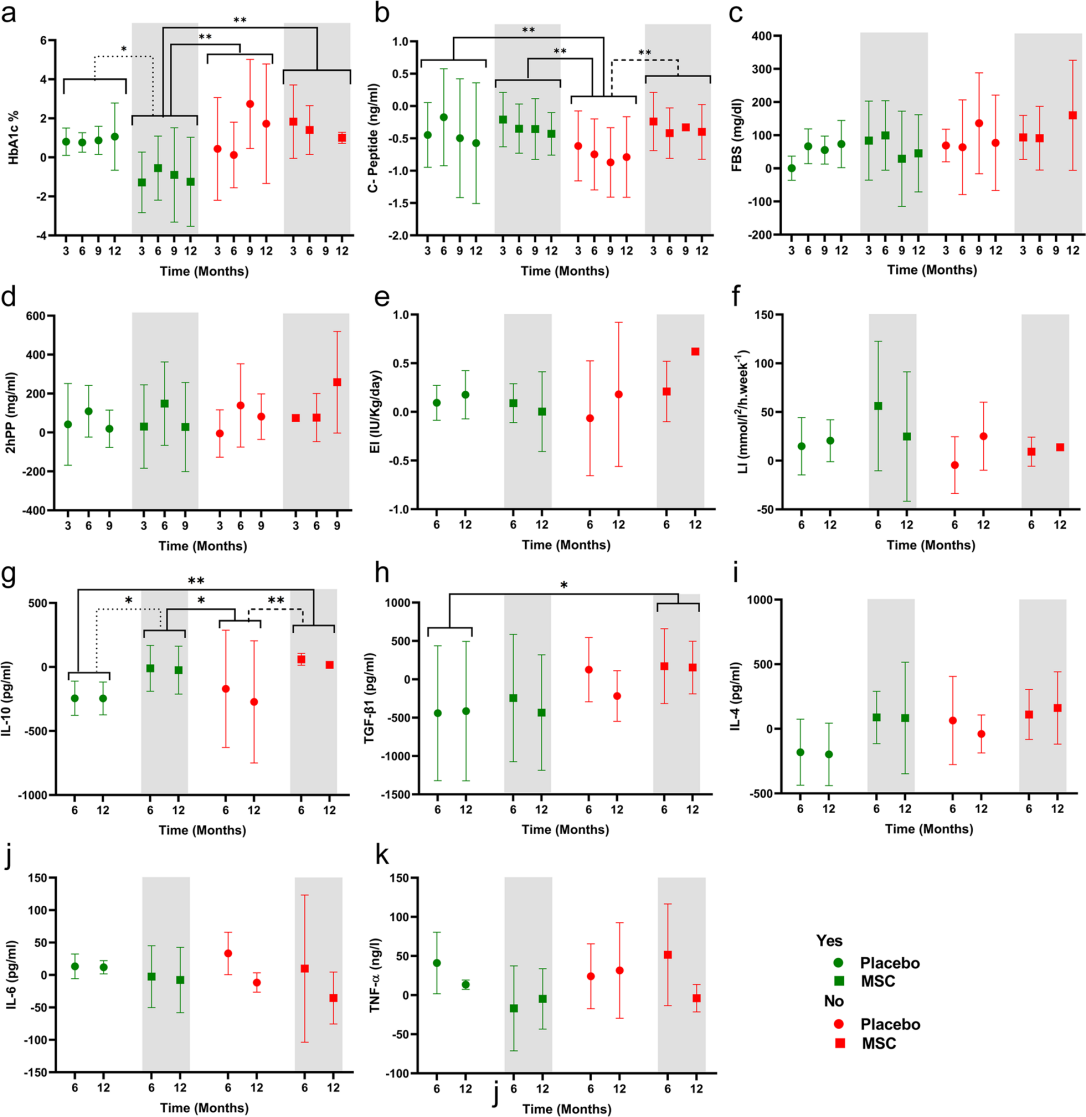
Comparison of metabolic indices and cytokine levels in the mesenchymal stem cell (MSC) and placebo groups based on patient exercise. **a** Glycated hemoglobin (HbA1c), **b** C-peptide, **c** Fasting blood sugar (FBS), **d** Two-hour postprandial (2hpp), **e.** Exogenous insulin (EI), **f** Lability index (LI), **g** Interleukin 10 (IL-10), **k** Tumor necrosis factor-alpha (TNF-*α*), **i** Interleukin 4 (IL-4), **j** Interleukin 6 (IL-6), **h** Transforming growth factor-beta 1 (TGF-*β*1). Data are presented as mean ± standard deviation with an exercise cut-off of 3.5 h/week. Yes, means more exercise than the cut-off. No, means less exercise than the cut-off. Chart legend for the entire picture is shown in part (**a**). *P < 0.05, **P < 0.01

The percentage of HbA1c in patients who received MSCs and exercised more than the cut-off mentioned above (in Fig. 8 shown by “Yes”) was significantly lower than those who received MSCs or placebo and did not exercise (in Fig. 8 shown by “No”) based on ADA recommendations (*P* = 0.0035, *P* = 0.0033, respectively, Fig. 8a). HbA1c levels in patients who received MSCs and who had adequate exercise levels were significantly lower than the placebo group, who had adequate levels of exercise (*P* = 0.0117, Fig. 8a).

Exercise significantly increased the serum C-peptide level of patients who received the placebo and had adequate levels of exercise (Yes, Placebo) compared to the patients who received placebo and exercised less than the recommendations (No, Placebo) (Fig. 8b, *P*  = 0.0088). Moreover, patients in the MSC group who had adequate exercise levels (Yes, MSC group) had significantly higher C-peptide levels than the placebo group who exercised less (No, Placebo group). The No, MSC group had higher C-peptide levels compared to the No, Placebo group (*P* = 0.0016, 0.0019, respectively, Fig. 8b). The level of FBS, 2hpp, and EI did not change significantly (Fig. 8c-f).

IL-10 was significantly higher in patients who received MSC than the placebo group, regardless of the level of exercise (Yes, MSC vs. Yes, Placebo: *P* = 0.0156; Yes, MSC vs. No, Placebo: *P* = 0.0228; No, MSC vs. Yes, Placebo: *P* = 0.0070; No, MSC vs. No, Placebo: *P* = 0.0097; Fig. 8j). The No, MSC group had significantly higher TGF-*β*1 levels compared to the Yes, Placebo group (*P* = 0.0438, Fig. 8k). The changes for IL-6, TNF-*α*, and IL-4 were insignificant (Fig. 8a, b, e-i).

## Discussion

To the best of our knowledge, this is the first randomized triple-blinded, placebo-controlled clinical trial that assesses the safety and efficacy of transplanting autologous bone marrow-derived MSCs as a treatment for newly diagnosed T1D patients. We found that MSCs transplantation is safe in diabetic children and significantly reduces the number of hypoglycemic episodes. MSCs transplantation improved HbA1c and quality of life (QOL). Compared with previous studies, we evaluated immunologic indices, including IL-6, TNF-*α*, IL-4, IL-10, TGF-*β*1 levels, and regulatory T cells, in addition to general diabetes evaluation. We found that MSCs transplantation altered cytokine milieu from pro-inflammation to anti-inflammation, which was further confirmed with an increased number of regulatory T-cells in peripheral blood post-transplantation. Also, early transplantation of MSCs improved metabolic and immunologic outcomes, and exercise combined with MSCs transplantation improved glycemic and immunologic indices. Overall, we used a wide range of clinical data to validate our hypothesis. Our findings may lead to new strategies to target newly diagnosed T1D patients.

We selected bone marrow as the source of MSCs, as it is the most common source of MSCs for clinical trials and the first reported source of MSCs [49, 53]. The reported efficiency of MSCs isolation from BM is more than umbilical cord blood. Also, it has been shown that UCB-derived MSCs barely showed adipogenic differentiation potential compared to BM-MSCs, which are fully capable of differentiating to osteoblasts, chondrocytes, and adipocytes [54]. As this study was mainly designed to recruit children newly diagnosed with type-1 diabetes, bone marrow was the selected source for isolation of MSCs, since the proliferation capacity of Bone-marrow is higher in children.

Our results showed that transplantation of bone marrow-derived MSCs in diabetic children is safe and well-tolerated over the study follow-up period. We followed the safety indices for 24 months and found that this procedure’s long-term safety was acceptable (data are not shown). No major complications and transplantation-associated adverse events were reported, supported by previous studies harboring MSC transplantations for various diseases [49, 55–64].

Interestingly, we observed that the MSC transplantation could significantly reduce the number of hypoglycemic events in recipients as a major life-threatening complication of T1D in children. This effect was reported in a preclinical study of MSC transplantation for post-hepatectomy liver failure [65]. Additionally, for the first time, we showed that early transplantation of MSCs significantly reduced the rate of grade II hypoglycemic events in T1D patients compared to late transplantation of MSCs.

The efficacy of MSC transplantation in our study was favorable. We observed a significant reduction of HbA1c at month 12 along with its considerable reduction in month 9. There were higher, but not significant, serum C-peptide levels, a significant increase in serum levels of IL-4 and IL-10, and a slight increase in TGF-*β*1 after 12 months as anti-inflammatory cytokines. Besides, there was a significant reduction in serum TNF-*α* at month 6 and a considerable reduction at month 12, along with a moderate decrease in IL-6 levels at 12 months, as pro-inflammatory cytokines. Previous studies showed a decreased level of IL-4 in newly diagnosed T1D patients [66]. We showed that MSCs could increase serum IL-4 levels. The anti-inflammatory properties of IL-4 might prevent disease progression. We did not observe any significant differences in FBS levels between the groups, which was confirmed by previous studies likely attributed to fluctuations in FBS levels, which are influenced by many variables [11].

Although IL-10 has an anti-inflammatory effect that prevents inflammation as the main pathogenesis of autoimmune disorders, its role in modulating disease progression in T1D is debatable [67]. The results of several studies suggest a correlation between elevated IL-10 and disease attenuation [67–69]. The proposed mechanism of action for IL-10 mediated T1D regulation includes increasing the numbers of T reg cells and serum levels of T-helper 2-type cytokines (IL-4 and IL-10) while reducing serum levels of T-helper 1-type cytokines (IL-2 and IFN-γ) [70, 71].

Potential immunosuppressive effects of TGF-*β* are accepted in autoimmune diseases used against most immune cells through the development and peripheral differentiation of Tregs and induced expression of FOXP3 in the context of T1D [67, 72].

IL-6 is a pro-inflammatory cytokine that plays a role in autoimmune disease progression. Preclinical studies report a correlation between IL-6 production and *β*-cell destruction [67, 73, 74]. Chen et al. conducted a systematic review and concluded that a link existed between serum IL-6 levels and T1D [75]. An ongoing clinical trial aims to preserve *β* cells through an IL-6 blockade by tocilizumab administration (NCT02293837).

TNF-*α* is a pro-inflammatory cytokine produced by dendritic cells and macrophages; its effects on MHC-I upregulation results in accelerating *β*-cell death and initiation of T1D [76, 77]. Mastrandrea et al., in a randomized pilot study, have reported preservation of *β*-cell function in newly diagnosed T1D patients who received TNF blockers [46]. Krogvold et al. analyzed the pancreatic tissues of deceased donors who died at their diabetes onset and showed the restoration of insulin secretion after modulating islets’ diabetogenic microenvironment in vitro [78].

Of note is an increase in anti-inflammatory cytokines (TGF-*β*, IL-4, and IL-10) and a decrease in pro-inflammatory cytokines (TNF-*α* and IL-6) with the promotion of T reg cells, which has been reported in other studies as some of the immunomodulatory functions of MSCs. These findings align with our results [13, 36, 39, 42, 45, 51, 79–81].

We also observed an improved QOL in the patients in terms of significantly reduced DQOL-worry and improved SF36-Role-Physical evaluation at month 6 as well as SF36-General Health at month 12.

In 2013, Hu et al. conducted a randomized clinical trial to evaluate the effects of Wharton’s jelly-derived MSC injection in 29 new-onset T1D patients, with 15 patients in the treatment group and 14 patients in the control group. They observed higher C-peptide levels and improved glycemic control after two years of follow-up [11]. Carlsson et al. reported that systemic injection of autologous bone marrow-derived MSCs in nine patients compared to a control group could preserve or even increase C-peptide levels in response to a mixed-meal tolerance test (MMTT) during the first year [59]. Our data support the results of these studies.

Some studies showed that hyperglycemia is caused by reduced *β*-cell mass and *β*-cell dysfunction. Its extent varies between patients and is based on patient age and level of insulitis. More than 80% loss of beta-cell mass at the onset of T1D is a common finding in studies related to T1D pathogenesis, which suggested a preserved *β*-cell mass with up to 20% residual insulin-secreting cells. Some researchers have stated that *β*-cell mass might be underestimated due to insulin degranulation, which occurs in exhausted *β* cells [82, 83].

From this point of view, we evaluated the efficiency of early versus late transplantation of MSCs following the occurrence of the first clinical symptoms of T1D, in addition to comparing MSC and placebo groups.

We found that early transplantation of MSCs in newly diagnosed patients had benefits compared to late transplantation. These benefits included a significant decrease in HbA1c at months 3, 6, and 12; significantly higher C-peptide levels at months 3, 6, and 9; significantly lower level of TNF-*α* at 12 months and slightly lower level of IL-6 at month 6 as pro-inflammatory cytokines. There were significant increases in the anti-inflammatory cytokines IL-4, IL-10, and TGF-*β*1 at 12 months. We also observed significantly reduced QOL in terms of DQOL and SF36-Social functioning in the Early Tx group, likely due to the time (one year) passed from the onset of diabetes in the Late Tx group. The patients were more adapted to their disease situation and more stable than newly diagnosed patients.

Another factor that affects blood glucose management in T1D patients is the activity level. Regular exercise should be encouraged in people with insulin-dependent diabetes to decrease cardiovascular disease risk and improve QOL. In addition, mild exercise increases insulin action without influencing BMI. Although physical exercise should be undertaken in T1D patients with safety recommendations for blood glucose monitoring before and after exercise to prevent hypoglycemia or hyperglycemia, this challenge could be mostly overcome with a regular, anticipated exercise program [84, 85].

We found that more than 3.5 h/week of exercise in addition to MSC transplantation could significantly decrease HbA1c levels and increase C-peptide and Il-10 levels when compared to patients who did not have enough exercise and received the placebo. Furthermore, the effects of the MSC transplantation are superior to exercise alone since patients who did not have enough exercise but received MSC transplantation had significantly higher levels of TGF-*β*1 and IL-10 compared to the placebo group patients who had enough exercise.

This study had several limitations. First, a limited number of patients met the defined eligibility criteria, which caused a longer than expected recruitment process. T1D is a multifactorial disease probably influenced by patients’ lifestyle, socioeconomic status, stress level, exercise, diet, and other known and unknown factors that we did not consider. Thus, some studies are needed to assess the effects of these factors on disease progression. International Society for Pediatric and Adolescent Diabetes (ISPAD) noted the importance of partial remission in the early stages of type-1 diabetes following insulin treatment initiation, known as the honeymoon phase. This period reflects the partial recovery of *β*-cell with increased insulin secretion, in which the metabolic indices, notably HbA1c and C-peptide, could improve toward near normoglycemia condition [86, 87]. In this study, since all participants were enrolled in their early stage of diagnosis, their condition could be affected by the honeymoon phase, which could have obscured the MSC’s effects. Finally, regarding the possible reduced therapeutic potential of diabetics’ MSCs, further evaluation should be considered for allogeneic MSCs transplantation.

## Conclusions

In this study, we found that transplantation of MSCs in T1D patients is safe and tolerable during the follow-up period. MSC transplantation can improve some of the patients’ metabolic indices and modulate their immune responses. Early transplantation of MSCs can greatly improve metabolic indices and alleviate immune responses by increasing anti-inflammatory cytokines and decreasing pro-inflammatory cytokines compared to late transplantation of MSCs. We observed a noticeable pro-inflammatory to anti-inflammatory cytokine shift in addition to an increased T reg cell population, which provided additional evidence for the benefits of early transplantation of MSCs. However, a detailed study with different cell doses and repeated injections is required to achieve superior clinical results.

Although it was hard to control lifestyle variables in patients, we recommended that all study participants exercise by considering safety concerns, resulting in enhanced glycemic control in a limited number of patients who exercised more than 3.5 h/week. Therefore, controlling patients’ lifestyles in addition to MSC transplantation could improve patients’ responses to therapy, which should be evaluated in a greater number of participants.

A phase III study with a higher number of patients who receive multiple doses of MSC injections and longer follow-ups are needed to elucidate the therapeutic effects of MSCs in T1D and shed light on its molecular mechanisms and pathways behind this process.

We conclude that autologous bone marrow-derived MSC transplantation is a safe therapeutic choice for newly diagnosed labile T1D patients who have high blood glucose fluctuations and experience several episodes of hypoglycemia.

## Supplementary Information

**Additional file 1: Table S1**. Materials used in this study. **Table S2**. Analysis of the carryover effect. **Table S3**. List of follow up events. **Table S4**. Complete list of assessed adverse events. **Table S5**. Comparison of metabolic indices in a 12-month follow-up of mesenchymal stem cells (MSCs) versus placebo. **Table S6**. Comparison of immunologic indices in 12 months follow-up of MSCs versus placebo. **Table S7**. Comparing the quality of life (QOL) questionnaires scores in 12 months follow-up of MSCs versus Placebo. **Table S8**. Comparison of quality of life (QOL) questionnaire scores in 12 months of follow-up between the early (Early Tx) and late (Late Tx) transplantation groups. **Table S9**. Comparison of metabolic indices in 12 months of follow-up between early versus late transplantation of MSCs. **Table S10**. Comparison of immunologic indices in 12 months of follow-up for the early (Early Tx) and late (Late Tx) transplantation groups. **Figure S1**. Sample of cytogenetic report of MSCs. **Figure S2**. Sample of microbiological and bacterial endotoxin report. **Figure S3**. Sample of mycoplasma report. **Figure S4**. Flow Cytometric Analysis of Mesenchymal Stem Cells. **Figure S5**. Mesenchymal Stem Cells (MSCs) morphology and differentiation potential

## Data Availability

The data that supports the findings of this study are available in the supplementary material of this article.

## Abbreviations

2hpp: Two-hour postprandial glucose test
ADA: American Diabetes Association
ALP: Alkaline phosphatase
ALT: Alanine aminotransferase
ANOVA: Analysis of variances
AST: Aspartate aminotransferase
BM: Bone marrow
BMI: Body mass index
BP: Bodily pain
CD: Cluster of differentiation
CI: Confidence intervals
CMV: Cytomegalovirus
CONSORT: Consolidated standards of reporting trials
CTCAE v.5: Common Terminology Criteria for Adverse Events Version 5
DQOL: Diabetes QOL
Early Tx: Early Transplantation Group
EDTA: Ethylenediaminetetraacetic acid
EI: Exogenous insulin
EKG: Electrocardiogram
ELISA: Enzyme-linked immunosorbent assay
FBS: Fasting blood sugar
FOXP3: Forkhead box P3
GADA: Glutamic acid decarboxylase antibody
GEE: Generalized estimating equation
GH: General health
GMP: Good manufacturing practice
HbA1c: Glycated hemoglobin
HBV: Hepatitis B virus
HCV: Hepatitis C virus
HIV: Human immunodeficiency virus
HLA-DR: Human leukocyte Antigen-DR isotype
HSCs: Hematopoietic stem cells
HTLV: Human T-lymphotropic virus
IA-2A: Insulinoma associated-2 antibody
ICA: Islet cell antibody
IDF: International Diabetes Federation
IL-10: Interleukin 10
IL-4: Interleukin 4
IL-6: Interleukin 6
iPSCs: Induced pluripotent stem cells
IRCT: Iranian Registry of Clinical Trials
ISCT: International Society for Cellular Therapy
ISPAD: International Society for Pediatric and Adolescent Diabetes
IV: Intravenously
Late Tx: Late Transplantation Group
LI: Lability index
LLN: Lower limit of normal
MEM *α*: Minimum essential medium *α*
MH: Mental health
MHC: Major histocompatibility complex
MITT: Modified intention to treatment
MMTT: Mixed-meal tolerance test
MSCs: Mesenchymal stem cells
NK cells: Natural killer cells
PBMCs: Peripheral blood mononuclear cells
PF: Physical functioning
QOL: Quality of life
RE: Role—emotional
RP: Role—physical
SF: Social functioning
SF-36: 36-Item short form survey
T reg: Regulatory T cells
T1D: Type-1 diabetes
TGF-*β*1: Transforming growth factor-beta 1
TNF-*α*: Tumor necrosis factor-alpha
UCB: Umbilical cord blood
VT: Vitality
