# Supplementary material for: Mesenchymal stem cell transplantation in newly diagnosed type-1 diabetes patients: a phase I/II randomized placebo-controlled clinical trial
Source: Stem Cell Res Ther. 2022 Jun 20;13:264. doi: 10.1186/s13287-022-02941-w (PMC9208234; doi:10.1186/s13287-022-02941-w)
Supplement: Supplementary file 1 — Additional file 1: Table S1. Materials used in this study. Table S2. Analysis of the carryover effect. Table S3. List of follow up events. Table S4. Complete list of assessed adverse events. Table S5. Comparison of metabolic indices in a 12-month follow-up of mesenchymal stem cells (MSCs) versus placebo. Table S6. Comparison of immunologic indices in 12 months follow-up of MSCs versus placebo. Table S7. Comparing the quality of life (QOL) questionnaires scores in 12 months follow-up of MSCs versus Placebo. Table S8. Comparison of quality of life (QOL) questionnaire scores in 12 months of follow-up between the early (Early Tx) and late (Late Tx) transplantation groups. Table S9. Comparison of metabolic indices in 12 months of follow-up between early versus late transplantation of MSCs. Table S10. Comparison of immunologic indices in 12 months of follow-up for the early (Early Tx) and late (Late Tx) transplantation groups. Figure S1. Sample of cytogenetic report of MSCs. Figure S2. Sample of microbiological and bacterial endotoxin report. Figure S3. Sample of mycoplasma report. Figure S4. Flow Cytometric Analysis of Mesenchymal Stem Cells. Figure S5. Mesenchymal Stem Cells (MSCs) morphology and differentiation potential [file 13287_2022_2941_MOESM1_ESM.docx]

Supplementary information

Mesenchymal Stem Cell Transplantation in Newly Diagnosed Type-1 Diabetes Patients: A Phase I/II Randomized Placebo Controlled Clinical Trial

Mahmoud Izadi, Anavasadat Sadr Hashemi Nejad, Maedeh Moazenchi, Safdar Masoumi, Ali Rabbani, Farzad Kompani, Amir Abbas Hedayati Asl, Fatemeh Abbasi Kakroodi, Neda Jaroughi, Mohammad Ali Mohseni Meybodi, Aria Setoodeh, Farzaneh Abbasi, Seyedeh Esmat Hosseini, Fatemeh Moeini Nia, Reza Salman Yazdi, Roghayeh Navabi, Ensiyeh Hajizadeh-Saffar, Hossein Baharvand

| **Table S1.** Materials used in this study | | | |
| --- | --- | --- | --- |
| **No.** | **Name** | **Manufacturer** | **Product number** |
| **Reagents** | | | |
| 1 | Ficoll density gradient | PAN biotech | P04-60100 |
| 2 | CliniMACS PBS/EDTA Buffer | Miltenyi Biotec GmbH | 700-25 |
| 3 | Trypsin - EDTA | Gibco | 25300054 |
| 4 | Lymphodex | Inno-Train | D-61476 |
| 5 | MEM α (Minimum Essential Medium α) | Gibco | 22571020 |
| 6 | Penicillin-Streptomycin (5,000 U/mL) | Gibco | 15070063 |
| 7 | HyClone Defined FBS, US Origin | Thermo Scientific | SH30070.03 |
| 8 | L-Glutamine | Gibco | 25030-024 |
| 9 | PBS | Gibco | 21600-051 |
| **Antibodies** | | | |
| 1 | FITC conjugated anti-CD4 | BD Biosciences | 550628 |
| 2 | PE-conjugated anti-CD25 | BD Biosciences | 557138 |
| 3 | PE Mouse anti-Human CD105 | BD Biosciences | 560839 |
| 4 | PE Mouse Anti-Human CD44 | BD Biosciences | 555479 |
| 5 | PE Mouse Anti-Human CD73 | BD Biosciences | 550257 |
| 6 | Mouse Anti-Human CD90/FITC | Dako | F7274 |
| 7 | CD34/CD45 Antibody Cocktail, FITC, PE | Thermo Fisher Scientific | MA1-12221 |
| 8 | CD44 Monoclonal Antibody (IM7), APC-eFluor 780, eBioscience™ | Thermo Fisher Scientific | 47-0441-82 |
| 9 | CD11b Monoclonal Antibody (ICRF44), PE, eBioscience™ | Thermo Fisher Scientific | 12-0118-42 |
| 10 | Cytofix/Cytoperm | BD Biosciences | 554722 |
| 11 | PerCP anti-Foxp3 antibody | Novus Biologicals | NBP2-34432PCP |
| 12 | BD Perm/Wash buffer | BD Biosciences | 554723 |
| **Kits** | | | |
| 1 | GLUC3 kit | Roche Diagnostics GmbH | 4404483190 |
| 2 | Elecsys C-peptide kit | Roche Diagnostics GmbH | 3184897 |
| 3 | Tina-quant® HbA1c Gen. 3 | Roche Diagnostics GmbH | 5336163190 |
| 4 | Human IL-6 ELISA Kit | Bioassay Technology Laboratory | E0090Hu |
| 5 | Human TNF-α ELISA Kit | Bioassay Technology Laboratory | E0082Hu |
| 6 | Human IL-4 Quantikine ELISA Kit | R&D Systems | D4050 |
| 7 | Human IL-10 Quantikine ELISA Kit | R&D Systems | D100B |
| 8 | Human TGF- β1 Quantikine ELISA Kit | R&D Systems | DB100B |

| Table S2. Analysis of the carryover effect^*^ | | | | | |
| --- | --- | --- | --- | --- | --- |
| Outcome | **Group**  $\beta_{1} (SE)$ | **Time**  $\beta_{2} (SE)$ | **Period**  $\beta_{3}(SE)$ | **Group * Period**  $\beta_{4} (SE)$ | **Carryover effect** |
| HbA1C (%) | 0.26 (0.53),  p=62 | 0.02 (0.2),  p=34 | 0.30 (0.55),  p=58 | 0.36 (0.76),  p=63 | No |
| FBS (mg/dl) | 41.1 (27.9), p=0.14 | 4.6 (1.4), p=0.001 | 34.2 (29.4), p=0.24 | -92.3 (40.9), **p=0.024** | **Yes** |
| 2hpp (mg/dl) | 66.1 (39.5), p=0.09 | 3.7 (2.1),  p=0.07 | 30.9 (42.0), p=0.74 | -84.2 (58.3), p=15 | No |
| C-peptide (ng/ml) | -0.04 (0.1), p=0.64 | -0.04 (0.01), p<0.001 | -0.18 (0.11), p=0.09 | 0.04 (0.15), p=0.82 | No |
| EI (IU/kg/day) | -0.01 (0.12), p=0.92 | 0.01(0.01), p=0.005 | 0.02 (0.13),  p=88 | 0.18 (0.18), p=0.31 | No |
| LI | -8.7 (13.64), p=0.52 | 1.24 (0.80), p=0.12 | -1.9 (15.1), p=0.90 | -4.5 (20.5), p=0.83 | No |
| TNF-α (ng/l) | 1.2 (12.25), p=0.92 | 2.86 (0.74), p=0.00 | 45.9 (13. 6), p=0.00 | -39.1 (18.6), **p=0.04** | **Yes** |
| IL-10 (pg/ml) | -56.2 (78.2), p=0.47 | -13.8 (3.8), p=0.00 | -135.96 (85.0), p=0.11 | 86.7 (116.3), p=0.46 | No |
| IL-4 (pg/ml) | 60.7 (94.9), p=0.52 | -3.7(4.2), p=0.38 | -1.62 (102.4), p=0.99 | -147.6 (140.3), p=0.29 | No |
| TGF-β (pg/ml) | -105.3 (173.8), p=0.55 | -23.6 (9.6), p=0.01 | -383.8 (191.6), p=0.05 | 143.9 (261.5), p=0.58 | No |
| IL-6 (ng/ l) | 4.6 (10.2), p=0.65 | -1.04 (0.95), p=0.27 | 20.2 (15.2), p=0.18 | -20.3 (19.4), p=0.30 | No |
| ^*^ The following model can be used to describe the crossover design:  *Y_ij_ =β_0_ + β_1_*Group+ β_2_*Time + β_3_*Period + β_4_*Group*Period*  The carryover effect, which corresponds to *β_4_* in the equation, refers to the effect of the previous treatment or the change caused by the first treatment continues until the next period and alters the effect of the subsequent treatment. Analyzing the period-by-treatment interaction determines whether the two treatment effects are different in the two periods. The carryover effect and the period-by-treatment interaction are often treated as identical. However, depending on the parameters included in the crossover design model, the carryover effect may be embedded in parameters other than the period-by-treatment interaction. | | | | | |

| **Table S3.** List of follow up events | | | | | | | | | | | | | |
| --- | --- | --- | --- | --- | --- | --- | --- | --- | --- | --- | --- | --- | --- |
| **Timepoint** | | **Hematology** | **Autoantibodies** | **Cytokines** | **Beta-cell function** | | **Liver function** | **Kidney function** | **QOL questionnaire** | **Other** | | | **Transplantation** |
|  |  | CBC diff, ESR, Na, K, Ca | ICA, GADA, IA-2A | TGF-β, IL-4, IL-10, TNF-α, IL-6 | FBS, Serum C-peptide | HbA1c,2hpp | AST, ALT, ALP | BUN, Cr | SF-36, DSQOL | ECG, chest X-ray | ESR, CRP, Na, K, Ca, HDL, LDL, Cholesterol, TG, U/A, U/C, T3, T4, TSH, PT, PTT, INR, Hbs Ag, Anti Hbs, Anti HBC, Anti HCV, Anti-HIV, Anti CMV, Anti HTLV I-II | Physician’s Visit |  |
| Entrance | |  | 🗸 |  | 🗸 |  |  |  |  |  |  |  |  |
| First visit | | 🗸 |  | 🗸 |  | 🗸 | 🗸 | 🗸 | 🗸 | 🗸 | 🗸 | 🗸 |  |
| Day 0 | |  |  |  |  |  |  |  |  |  |  |  | 🗸 |
| Week 1 | |  |  |  | 🗸 |  |  |  |  |  |  | 🗸 |  |
| Week 2 | |  |  |  | 🗸 |  |  |  |  |  |  |  |  |
| Week 3 | |  |  |  | 🗸 |  |  |  |  |  |  |  | 🗸 |
| Month 1 | |  |  |  | 🗸 |  |  |  |  |  |  | 🗸 |  |
| Month 2 | |  |  |  | 🗸 |  |  |  |  |  |  |  |  |
| Month 3 | |  |  |  | 🗸 | 🗸 |  |  |  |  |  | 🗸 |  |
| Month 6 | | 🗸 | 🗸 | 🗸 | 🗸 | 🗸 | 🗸 | 🗸 | 🗸 | 🗸 |  | 🗸 |  |
| Month 9 | |  |  |  | 🗸 | 🗸 |  |  |  |  |  | 🗸 |  |
| Month 12 |  | 🗸 | 🗸 | 🗸 | 🗸 | 🗸 | 🗸 | 🗸 | 🗸 | 🗸 |  | 🗸 | 🗸 |
|  | + 1 week |  |  |  | 🗸 |  |  |  |  |  |  |  |  |
|  | +2 week |  |  |  | 🗸 |  |  |  |  |  |  |  |  |
|  | + 3 week |  |  |  | 🗸 |  |  |  |  |  |  |  | 🗸 |
| Month 13 | |  |  |  | 🗸 |  |  |  |  |  |  |  |  |
| Month 14 | |  |  |  | 🗸 |  |  |  |  |  |  |  |  |
| Month 15 | |  |  |  | 🗸 | 🗸 |  |  |  |  |  | 🗸 |  |
| Month 18 | |  |  |  | 🗸 | 🗸 |  |  | 🗸 |  |  | 🗸 |  |
| Month 21 | |  |  |  | 🗸 | 🗸 |  |  |  |  |  |  |  |
| Month 24 | | 🗸 | 🗸 | 🗸 | 🗸 | 🗸 | 🗸 | 🗸 | 🗸 | 🗸 |  | 🗸 |  |

| **Table S4.** Complete list of assessed adverse events. | | | | | | | |
| --- | --- | --- | --- | --- | --- | --- | --- |
| **No.** | **Med DRA Code** | **CTCAE Term** | **Observed Grade** | **Case** | | **Placebo** | |
|  |  |  |  | N Patients | N Events | N Patients | N Events |
| **IMMUNE SYSTEM DISORDER** | | | | | | | |
| 1 | 10001718 | Allergic reaction | **1** |  | 0 |  | 0 |
|  |  |  | **2** |  | 0 |  | 0 |
|  |  |  | **3** |  | 0 |  | 0 |
|  |  |  | **4** |  | 0 |  | 0 |
|  |  |  | **5** |  | 0 |  | 0 |
| **BLOOD AND LYMPHATIC SYSTEM DISORDER** | | | | | | | |
| 2 | 10024378 | Leukocytosis | **1** |  | 0 |  | 0 |
|  |  |  | **2** |  | 0 |  | 0 |
|  |  |  | **3** |  | 0 |  | 0 |
|  |  |  | **4** |  | 0 |  | 0 |
|  |  |  | **5** |  | 0 |  | 0 |
| **INVESTIGATIONS** | | | | | | | |
| 3 | 10000636 | Activated partial thromboplastin time prolonged | **1** |  | 0 |  | 0 |
|  |  |  | **2** |  | 0 |  | 0 |
|  |  |  | **3** |  | 0 |  | 0 |
|  |  |  | **4** |  | 0 |  | 0 |
|  |  |  | **5** |  | 0 |  | 0 |
| 4 | 10001551 | Alanine aminotransferase (ALT) increased | **1** |  | 0 | 1 | 1 |
|  |  |  | **2** |  | 0 |  | 0 |
|  |  |  | **3** |  | 0 |  | 0 |
|  |  |  | **4** |  | 0 |  | 0 |
|  |  |  | **5** |  | 0 |  | 0 |
| 5 | 10001675 | Alkaline phosphatase (ALP) increased | **1** | 3 | 5 | 3 | 4 |
|  |  |  | **2** |  | 0 |  | 0 |
|  |  |  | **3** |  | 0 |  | 0 |
|  |  |  | **4** |  | 0 |  | 0 |
|  |  |  | **5** |  | 0 |  | 0 |
| 6 | 10003481 | Aspartate aminotransferase (AST) increased | **1** |  | 0 | 2 | 2 |
|  |  |  | **2** |  | 0 |  | 0 |
|  |  |  | **3** |  | 0 |  | 0 |
|  |  |  | **4** |  | 0 |  | 0 |
|  |  |  | **5** |  | 0 |  | 0 |
| 7 | 10008661 | Cholesterol high | **1** |  | 0 |  | 0 |
|  |  |  | **2** |  | 0 |  | 0 |
|  |  |  | **3** |  | 0 |  | 0 |
|  |  |  | **4** |  | 0 |  | 0 |
|  |  |  | **5** |  | 0 |  | 0 |
| 8 | 10011368 | Creatinine increased | **1** |  | 0 |  | 0 |
|  |  |  | **2** |  | 0 |  | 0 |
|  |  |  | **3** |  | 0 |  | 0 |
|  |  |  | **4** |  | 0 |  | 0 |
|  |  |  | **5** |  | 0 |  | 0 |
| 9 | 10014383 | Electrocardiogram QT corrected interval prolonged | **1** |  | 0 |  | 0 |
|  |  |  | **2** |  | 0 |  | 0 |
|  |  |  | **3** |  | 0 |  | 0 |
|  |  |  | **4** |  | 0 |  | 0 |
|  |  |  | **5** |  | 0 |  | 0 |
| 10 | 10022402 | INR increased | **1** |  | 0 |  | 0 |
|  |  |  | **2** |  | 0 |  | 0 |
|  |  |  | **3** |  | 0 |  | 0 |
|  |  |  | **4** |  | 0 |  | 0 |
|  |  |  | **5** |  | 0 |  | 0 |
| 11 | 10025258 | Lymphocyte count increased | **1** |  | 0 |  | 0 |
|  |  |  | **2** | 1 | 1 |  | 0 |
|  |  |  | **3** |  | 0 |  | 0 |
|  |  |  | **4** |  | 0 |  | 0 |
|  |  |  | **5** |  | 0 |  | 0 |
| 12 | 10025256 | Lymphocyte count decreased | **1** |  | 0 |  | 0 |
|  |  |  | **2** |  | 0 |  | 0 |
|  |  |  | **3** |  | 0 |  | 0 |
|  |  |  | **4** |  | 0 |  | 0 |
|  |  |  | **5** |  | 0 |  | 0 |
| 13 | 10029366 | Neutrophil count decreased | **1** |  | 0 |  | 0 |
|  |  |  | **2** |  | 0 |  | 0 |
|  |  |  | **3** |  | 0 |  | 0 |
|  |  |  | **4** |  | 0 |  | 0 |
|  |  |  | **5** |  | 0 |  | 0 |
| 14 | 10035528 | Platelet count decreased | **1** |  | 0 |  | 0 |
|  |  |  | **2** |  | 0 |  | 0 |
|  |  |  | **3** |  | 0 |  | 0 |
|  |  |  | **4** |  | 0 |  | 0 |
|  |  |  | **5** |  | 0 |  | 0 |
| 15 | 10047900 | Weight loss | **1** |  | 0 | 1 | 1 |
|  |  |  | **2** |  | 0 |  | 0 |
|  |  |  | **3** |  | 0 |  | 0 |
|  |  |  | **4** |  | 0 |  | 0 |
|  |  |  | **5** |  | 0 |  | 0 |
| **RENAL AND URINARY DISORDERS** | | | | | | | |
| 16 | 10019489 | Hemoglobinuria | **1** |  | 0 |  | 0 |
|  |  |  | **2** |  | 0 |  | 0 |
|  |  |  | **3** |  | 0 |  | 0 |
|  |  |  | **4** |  | 0 |  | 0 |
|  |  |  | **5** |  | 0 |  | 0 |
| **CARDIAC DISORDER** | | | | | | | |
| 17 | 10003673 | Atrioventricular block complete | **1** |  | 0 |  | 0 |
|  |  |  | **2** |  | 0 |  | 0 |
|  |  |  | **3** |  | 0 |  | 0 |
|  |  |  | **4** |  | 0 |  | 0 |
|  |  |  | **5** |  | 0 |  | 0 |
| 18 | 10003674 | Atrioventricular block first degree | **1** |  | 0 |  | 0 |
|  |  |  | **2** |  | 0 |  | 0 |
|  |  |  | **3** |  | 0 |  | 0 |
|  |  |  | **4** |  | 0 |  | 0 |
|  |  |  | **5** |  | 0 |  | 0 |
| 19 | 10033557 | Palpitations | **1** |  | 0 |  | 0 |
|  |  |  | **2** |  | 0 |  | 0 |
|  |  |  | **3** |  | 0 |  | 0 |
|  |  |  | **4** |  | 0 |  | 0 |
|  |  |  | **5** |  | 0 |  | 0 |
| 20 | 10042604 | Supraventricular tachycardia | **1** |  | 0 |  | 0 |
|  |  |  | **2** |  | 0 |  | 0 |
|  |  |  | **3** |  | 0 |  | 0 |
|  |  |  | **4** |  | 0 |  | 0 |
|  |  |  | **5** |  | 0 |  | 0 |
| **GENERAL DISORDERS AND ADMINISTRATION SITE CONDITIONS** | | | | | | | |
| 21 | 10008531 | Chills | **1** |  | 0 |  | 0 |
|  |  |  | **2** |  | 0 |  | 0 |
|  |  |  | **3** |  | 0 |  | 0 |
|  |  |  | **4** |  | 0 |  | 0 |
|  |  |  | **5** |  | 0 |  | 0 |
| 22 | 10016256 | Fatigue | **1** |  | 0 |  | 0 |
|  |  |  | **2** |  | 0 |  | 0 |
|  |  |  | **3** |  | 0 |  | 0 |
|  |  |  | **4** |  | 0 |  | 0 |
|  |  |  | **5** |  | 0 |  | 0 |
| 23 | 10016558 | Fever | **1** |  | 0 | 1 | 1 |
|  |  |  | **2** |  | 0 |  | 0 |
|  |  |  | **3** |  | 0 |  | 0 |
|  |  |  | **4** |  | 0 |  | 0 |
|  |  |  | **5** |  | 0 |  | 0 |
| 24 | 10021113 | Hypothermia | **1** |  | 0 |  | 0 |
|  |  |  | **2** |  | 0 |  | 0 |
|  |  |  | **3** |  | 0 |  | 0 |
|  |  |  | **4** |  | 0 |  | 0 |
|  |  |  | **5** |  | 0 |  | 0 |
| 25 | 10022095 | Injection site reaction | **1** | 1 | 1 | 1 | 1 |
|  |  |  | **2** | 1 | 1 | 1 | 1 |
|  |  |  | **3** |  | 0 |  | 0 |
|  |  |  | **4** |  | 0 |  | 0 |
|  |  |  | **5** |  | 0 |  | 0 |
| 26 | 10033371 | Pain (Epigastric region) | **1** | 1 | 1 | 1 | 1 |
|  |  |  | **2** |  | 0 |  | 0 |
|  |  |  | **3** |  | 0 |  | 0 |
|  |  |  | **4** |  | 0 |  | 0 |
|  |  |  | **5** |  | 0 |  | 0 |
| **METABOLISM AND NUTRITION DISORDERS** | | | | | | | |
| 27 | 10020587 | Hypercalcemia | **1** |  | 0 | 1 | 1 |
|  |  |  | **2** |  | 0 |  | 0 |
|  |  |  | **3** |  | 0 |  | 0 |
|  |  |  | **4** |  | 0 |  | 0 |
|  |  |  | **5** |  | 0 |  | 0 |
| 28 | 10020647 | Hyperkalemia | **1** |  | 0 |  | 0 |
|  |  |  | **2** |  | 0 |  | 0 |
|  |  |  | **3** |  | 0 |  | 0 |
|  |  |  | **4** | 1 | 1 |  | 0 |
|  |  |  | **5** |  | 0 |  | 0 |
| 29 | 10020680 | Hypernatremia | **1** |  | 0 |  | 0 |
|  |  |  | **2** |  | 0 |  | 0 |
|  |  |  | **3** |  | 0 |  | 0 |
|  |  |  | **4** |  | 0 |  | 0 |
|  |  |  | **5** |  | 0 |  | 0 |
| 30 | 10020870 | Hypertriglyceridemia | **1** |  | 0 |  | 0 |
|  |  |  | **2** |  | 0 |  | 0 |
|  |  |  | **3** |  | 0 |  | 0 |
|  |  |  | **4** |  | 0 |  | 0 |
|  |  |  | **5** |  | 0 |  | 0 |
| 31 | 10020907 | Hyperuricemia | **1** |  | 0 | 1 | 1 |
|  |  |  | **2** |  | 0 |  | 0 |
|  |  |  | **3** |  | 0 |  | 0 |
|  |  |  | **4** |  | 0 |  | 0 |
|  |  |  | **5** |  | 0 |  | 0 |
| 32 | 10020949 | Hypocalcemia | **1** |  | 0 |  | 0 |
|  |  |  | **2** |  | 0 | 1 | 1 |
|  |  |  | **3** |  | 0 |  | 0 |
|  |  |  | **4** |  | 0 |  | 0 |
|  |  |  | **5** |  | 0 |  | 0 |
| 33 | 10021005 | Hypoglycemia | **1** | 8 | 206 | 9 | 504 |
|  |  |  | **2** | 5 | 38 | 9 | 69 |
|  |  |  | **3** | 2 | 3 | 3 | 3 |
|  |  |  | **4** | 1 | 1 | 2 | 4 |
|  |  |  | **5** |  | 0 |  | 0 |
| 34 | 10021018 | Hypokalemia | **1** |  | 0 | 1 | 1 |
|  |  |  | **2** |  | 0 |  | 0 |
|  |  |  | **3** |  | 0 |  | 0 |
|  |  |  | **4** |  | 0 |  | 0 |
|  |  |  | **5** |  | 0 |  | 0 |
| 35 | 10021038 | Hyponatremia | **1** |  | 0 | 2 | 2 |
|  |  |  | **2** |  | 0 |  | 0 |
|  |  |  | **3** |  | 0 |  | 0 |
|  |  |  | **4** |  | 0 |  | 0 |
|  |  |  | **5** |  | 0 |  | 0 |
| **SKIN AND SUBCUTANEOUS TISSUE DISORDER** | | | | | | | |
| 36 | 10037847 | Rash acneiform | **1** |  | 0 |  | 0 |
|  |  |  | **2** |  | 0 |  | 0 |
|  |  |  | **3** |  | 0 |  | 0 |
|  |  |  | **4** |  | 0 |  | 0 |
|  |  |  | **5** |  | 0 |  | 0 |
| 37 | 10037868 | Rash maculopapular | **1** |  | 0 |  | 0 |
|  |  |  | **2** |  | 0 |  | 0 |
|  |  |  | **3** |  | 0 |  | 0 |
|  |  |  | **4** |  | 0 |  | 0 |
|  |  |  | **5** |  | 0 |  | 0 |
| 38 | 10046735 | Urticaria | **1** |  | 0 |  | 0 |
|  |  |  | **2** |  | 0 |  | 0 |
|  |  |  | **3** | 1 | 1 |  | 0 |
|  |  |  | **4** |  | 0 |  | 0 |
|  |  |  | **5** |  | 0 |  | 0 |
| **ENDOCRINE** | | | | | | | |
| 39 | 10020850 | Hyperthyroidism | **1** |  | 0 |  | 0 |
|  |  |  | **2** |  | 0 |  | 0 |
|  |  |  | **3** |  | 0 |  | 0 |
|  |  |  | **4** |  | 0 |  | 0 |
|  |  |  | **5** |  | 0 |  | 0 |
| 40 | 10021114 | Hypothyroidism | **1** |  | 0 | 1 | 1 |
|  |  |  | **2** |  | 0 |  | 0 |
|  |  |  | **3** |  | 0 |  | 0 |
|  |  |  | **4** |  | 0 |  | 0 |
|  |  |  | **5** |  | 0 |  | 0 |
| **GASTROINTESTINAL DISORDERS** | | | | | | | |
| 41 | 10000060 | Abdominal distension | **1** |  | 0 |  | 0 |
|  |  |  | **2** |  | 0 |  | 0 |
|  |  |  | **3** |  | 0 |  | 0 |
|  |  |  | **4** |  | 0 |  | 0 |
|  |  |  | **5** |  | 0 |  | 0 |
| 42 | 10010774 | Constipation | **1** |  | 0 |  | 0 |
|  |  |  | **2** |  | 0 |  | 0 |
|  |  |  | **3** |  | 0 |  | 0 |
|  |  |  | **4** |  | 0 |  | 0 |
|  |  |  | **5** |  | 0 |  | 0 |
| 43 | 10012727 | Diarrhea | **1** |  | 0 |  | 0 |
|  |  |  | **2** |  | 0 |  | 0 |
|  |  |  | **3** |  | 0 |  | 0 |
|  |  |  | **4** |  | 0 |  | 0 |
|  |  |  | **5** |  | 0 |  | 0 |
| 44 | 10028813 | Nausea | **1** |  | 0 |  | 0 |
|  |  |  | **2** |  | 0 |  | 0 |
|  |  |  | **3** |  | 0 |  | 0 |
|  |  |  | **4** |  | 0 |  | 0 |
|  |  |  | **5** |  | 0 |  | 0 |
| 45 | 10047700 | Vomiting | **1** |  | 0 |  | 0 |
|  |  |  | **2** |  | 0 |  | 0 |
|  |  |  | **3** |  | 0 |  | 0 |
|  |  |  | **4** |  | 0 |  | 0 |
|  |  |  | **5** |  | 0 |  | 0 |
| **INFECTIONS AND INFESTATIONS** | | | | | | | |
| 46 | 10021881 | Infections and infestations - Other, specify | **1** |  | 0 |  | 0 |
|  |  |  | **2** |  | 0 |  | 0 |
|  |  |  | **3** |  | 0 |  | 0 |
|  |  |  | **4** |  | 0 |  | 0 |
|  |  |  | **5** |  | 0 |  | 0 |
| **NEUROLOGY** | | | | | | | |
| 47 | 10037175 | Psychiatric disorders - Other, specify | **1** |  | 0 | 1 | 1 |
|  |  |  | **2** |  | 0 |  | 0 |
|  |  |  | **3** |  | 0 |  | 0 |
|  |  |  | **4** |  | 0 |  | 0 |
|  |  |  | **5** |  | 0 |  | 0 |

| **Table S5.** Comparison of metabolic indices in a 12-month follow-up of mesenchymal stem cells (MSCs) versus placebo. | | | | | | | | | | | | |  |
| --- | --- | --- | --- | --- | --- | --- | --- | --- | --- | --- | --- | --- | --- |
|  | Test of within-group effects^*^  (mean change from baseline) | | | | | | Test of between-group effects^*^  (mean change from reference group) | | | | | |  |
| **Outcomes/ Time (month)** | **Placebo (n=10)** |  | **MSCs (n=11)** | | | | | **MSCs vs. placebo** | | | | | |
|  | **Mean (95% CI)** | **Mean (95% CI)** | | |  |  | **MD** | |  | **95% CI** |  | **P-value** |  |
| FBS (mg/dl) | |  | | |  |  |  | |  |  |  |  |  |
| 3 | 44.4 (-17.0,105.8) | 88.7 (17.3,160.1) | | |  |  | 44.32 | | (-50.40, 139.06) | | | 0.359 |  |
| 6 | 61.3 (-0.14, 122.7) | 84.6 (13.2,156.0) | | |  |  | 23.33 | | (-71.39, 118.07) | | | 0.629 |  |
| 9 | 60.4 (-8.4, 129.2) | 72.4 (-6.0,150.8) | | |  |  | 12.24 | | (-93.05, 117.55) | | | 0.820 |  |
| 12 | 63.5 (1.7, 125.23) | 87.2 (15.8,158.0) | | |  |  | 23.68 | | (-71.05, 118.41) | | | 0.624 |  |
| F^a^ (%) | 49.6 (-3.3, 102.6) | 96.3 (10.9, 181.7) | | |  |  |  | |  |  |  |  |  |
| 2hpp (mg/dl) | |  | | |  |  |  | |  |  |  |  |  |
| 3 | -18.2 (-105.8, 69.4) | 58.6 (-53.7,170.9) | | |  |  | 73.56 | |  | (-74.0, 221.1) |  | 0.329 |  |
| 6 | 89.0 (11.0, 166.9) | 99.7 (-12.6, 211.9) | | |  |  | 10.83 | |  | (-127.8, 149.5) |  | 0.878 |  |
| 12 | 35.9 (-42.0, 113.8) | 93.5 (-18.8, 205.8) | | |  |  | 57.75 | |  | (-80.9, 196.4) |  | 0.414 |  |
| F (%) | 21.5 (-13.2, 56.3) | 112.5 (2.4, 222.6) | | |  |  |  | |  |  |  |  |  |
| HbA1C (%) | |  | | |  |  |  | |  |  |  |  |  |
| 3 | 0.26 (-0.83,1.35) | 0.02 (-0.90,0.93) | | |  |  | -0.28 | |  | (-1.72, 1.15) |  | 0.699 |  |
| 6 | 0.31 (-0.68, 1.32) | 0.27 (-0.64, 1.18) | | |  |  | -0.03 | |  | (-1.40, 1.33) |  | 0.964 |  |
| 9 | 1.33 (0.23, 2.43) | -0.01 (-1.06,1.06) | | |  |  | -1.37 | |  | (-2.91, 0.16) |  | 0.061 |  |
| 12 | 0.97 (-0.07, 2.02) | -0.46 (-1.41,0.47) | | |  |  | -1.46 | |  | **(-2.88, -0.04)** |  | **0.043** |  |
| F (%) | 16.3 (-9.3, 41.9) | -2.2 (-14.9, 10.6) | | |  |  |  | |  |  |  |  |  |
| C-peptide (ng/ml) | |  | | |  |  |  | |  |  |  |  |  |
| 3 | -0.45 (-0.70, -0.20) | -0.15 (-0.34, 0.03) | | |  |  | 0.29 | |  | (-0.016, 0.60) |  | 0.063 |  |
| 6 | -0.43 (-0.68, -0.18) | -0.38 (-0.57, -0.19) | | |  |  | 0.05 | |  | (-0.25, 0.36) |  | 0.74 |  |
| 9 | -0.61 (-0.86, -0.36) | -0.40 (-0.60, -0.20) | | |  |  | 0.21 | |  | (-0.10, 0.53) |  | 0.182 |  |
| 12 | -0.61 (-0.86, -0.34) | -0.41 (-0.60, -0.22) | | |  |  | 0.20 | |  | (-0.11, 0.51) |  | 0.214 |  |
| F (%) | -57.7 (-79.8, -35.5) | -46.5 (-81.8, -11.2) | | |  |  |  | |  |  |  |  |  |
| EI (I.U./kg/day) | |  | | |  |  |  | |  |  |  |  |  |
| 6 | 0.15 (0.001, 0.29) | 0.02 (-0.24, 0.28) | | |  |  | -0.13 | |  | (-0.43, 0.17) |  | 0.394 |  |
| 12 | 0.01 (-0.12, 0.15) | 0.25 (-0.02, 0.53) | | |  |  | 0.23 | |  | (-0.07, 0.54) |  | 0.132 |  |
| F(%) | 8.62 (-19.37, 36.61) | 76.1 (1.62, 150.58) | | |  |  |  | |  |  |  |  |  |
| LI (mmol/l^2^/h.week^-1^) | |  | | |  |  |  | |  |  |  |  |  |
| 6 | -6.67 (-32.91, 19.55) | 57.64 (18.24, 97.04) | | | |  | 65.58 | |  | (-6.05, 7.29) |  | 0.856 |  |
| 12 | 16.29 (-11.12, 43.71) | 14.69 (-26.37, 55.77) | | | |  | -4.52 | |  | (-2.51, 9.02) |  | 0.269 |  |
| F (%) | 1016.24 (-633.2, 2665.7) | 336.9 (-456.8, 1130.7) | | | |  |  | |  |  |  |  |  |
| ^*^ 95% CIs and P-values were adjusted using a generalized estimating equation (GEE) with body mass index (BMI), age, and sex as confounders. | | | | | | | | | | | | |  |
| CI: Confidence interval  MD: Mean difference  Significant P-values are shown in **bold** type.  FBS: Fasting blood sugar  ^a^ (\|Baseline – month 12\| /Baseline) *100 | | | | 2hpp: Two-hour postprandial  HbA1c: Glycated hemoglobin  EI: Exogenous insulin  LI: Lability index | | | | | | | | |  |
|  | | | | | | | | | | | | |  |

| **Table S6.** Comparison of immunologic indices in 12 months follow-up of MSCs versus placebo. | | | | | | | | |
| --- | --- | --- | --- | --- | --- | --- | --- | --- |
|  | Test of within-group effects^*^  (mean change from baseline) | | |  | | Test of between-group effects^*^  (mean change from reference group) | | |
| **Outcome/time (month)** | **Placebo (n=10)** | **MSC (n=11)** | | |  | **MSC vs. Placebo** | | |
|  | **Mean (95% CI)** | **Mean (95% CI)** | | |  | **MD** | **95% CI** | **P-value** |
| **Pro-inflammatory** | |  | | |  |  |  |  |
| IL-6 (ng/l) | |  | | |  |  |  |  |
| 6 | 21.48 (3.4, 39.58) | -3.9 (-34.38, 26.57) | | |  | -25.39 | (-61.72, 10.93) | 0.171 |
| 12 | -6.42 (-24.53, 11.67) | -16.75 (-47.24, 13.72) | | |  | -10.33 | (-46.66, 25.99) | 0.577 |
| F^a^ (%) | -2.20 (-15.8, 11.46) | -6.37 (-34.5, 21.79) | | |  |  |  |  |
| TNF-α (ng/l) |  |  | | |  |  |  |  |
| 6 | 34.79 (12.27, 57.32) | -6.18 (-34.2, 21.85) | | |  | -40.98 | (-77.40, -4.56) | **0.027** |
| 12 | 27.62 (5.10,50.15) | -5.89 (-33.94, 22.14) | | |  | -33.52 | (-69.9, 2.89) | 0.071 |
| F (%) | 62.4 (20.7, 104.2) | 6.5 (-12.2, 25.30) | | |  |  |  |  |
| **Anti-inflammatory** | |  | | |  |  |  |  |
| IL-4 (pg/ml) | |  | | |  |  |  |  |
| 6 | -74.0 (-232.5 ,84.3) | 52.47 (-146.5, 251.5) | | |  | 126.55 | (-131.1, 384.2) | 0.336 |
| 12 | -119.5 (-277.9, 38.9) | 161.78 (-37.2, 360.8) | | |  | 281.31 | (23.6, 538.9) | **0.032** |
| F (%) | -42.2 (-72.6, -11.9) | 61.5 (-16.2, 139.3) | | |  |  |  |  |
| IL-10 (pg/ml) | |  | | |  |  |  |  |
| 6 | -153.5 (-325.1, 18.16) | 3.99 (-72.46, 80.46) | | |  | 157.50 | (-24.76, 339.77) | 0.090 |
| 12 | -194.3 (-366.0, -22.7) | 7.92 (-68.5, 84.38) | | |  | 202.28 | (-20.01, 384.55) | **0.030** |
| F (%) | -29.8 (-80.4, 20.6) | 12.8 (-33.2, 58.8) | | |  |  |  |  |
| TGF-β1 (pg/ml) | |  | | |  |  |  |  |
| 6 | -47.91 (-317.9, 222.1) | 43.01 (-531.4, 617.4) | | |  | 90.93 | (-564.27, 746.13) | 0.786 |
| 12 | -228.9 (-499.0, 41.06) | 152.1 (-422.3 ,726.6) | | |  | 381.11 | (-274.08, 1036.31) | 0.254 |
| F (%) | -30.0 (-71.5, 11.4) | 119.4 (-143.9, 382.7) | | |  |  |  |  |
| ^*^ CIs and P-values were adjusted using a generalized estimating equation (GEE) by body mass index (BMI), age, and sex as confounders. | | | | | | | | |
| CI: Confidence interval  MD: Mean difference  Significant P-values are shown in **bold** type.  IL-6: Interleukin 6  ^a^(\|Baseline – month 12\| / Baseline) *100 | | | MSCs: Mesenchymal stem cells  TNF-α: Tumor necrosis factor-alpha  IL-4: Interleukin 4  IL-10: Interleukin 10  TGF-β1: Transforming growth factor-beta 1 | | | | | |

| **Table S7.** Comparing the quality of life (QOL) questionnaires scores in 12 months follow-up of MSCs versus Placebo | | | | | | | | | | | | |  |
| --- | --- | --- | --- | --- | --- | --- | --- | --- | --- | --- | --- | --- | --- |
|  | Test of Within-group effects**^*^**  (mean change from baseline) | | | | | | Test of between-group effects**^*^**  (mean change from reference group) | | | | | |  |
| **Outcomes/ Time (Month)** | **Placebo (n=10)** |  | **MSC (n=11)** | | |  | | **MSC vs. Placebo** | | | | | |
|  | **Mean (95%CI)** | **Mean (95%CI)** | | |  |  | | **MD** |  | **95% CI** |  | **P-value** |  |
| **Total DQOL** | | | | | | | | | | |  |  |  |
| 6 | 1.06 (-3.82, 5.95) | -0.57 (-4.63, 5.77) | | |  |  | | -0.55 |  | (-7.78, 6.68) |  | 0.881 |  |
| 12 | -1.94 (-7.06, 3.16) | -4.98 (-10.81, 0.84) | | |  |  | | -3.43 |  | (-11.26, 4.39) |  | 0.390 |  |
| F^a^ (%) | -1.12 (-12.12, 9.87) | -5.32 (-17.34, 6.69) | | |  |  | |  |  |  |  |  |  |
| Satisfaction | |  | | |  |  | |  |  |  |  |  |  |
| 6 | 2.29 (-6.36, 10.97) | -1.58 (-10.09, 6.91) | | |  |  | | -3.89 |  | (-16.07, 8.29) |  | 0.531 |  |
| 12 | -7.17 (-16.20, 1.85) | -8.30 (-17.80, 1.20) | | |  |  | | -1.15 |  | (-14.29, 11.97) |  | 0.863 |  |
| F (%) | -7.13 (-30.03, 15.76) | 0.27 (-26.31, 26.86) | | | |  | |  |  |  |  |  |  |
| Impact | |  | | |  |  | |  |  |  |  |  |  |
| 6 | 2.10 (-4.04, 8.25) | -0.47 (-7.58, 6.64) | | |  |  | | -2.64 |  | (-12.22, 6.92) |  | 0.588 |  |
| 12 | 2.52 (-3.90, 8.95) | -5.10 (-13.04, 2.83) | | |  |  | | -8.18 |  | (-18.51, 2.14) |  | 0.121 |  |
| F (%) | 5.30 (-8.99, 19.59) | -8.72 (-26.27, 8.81) | | |  |  | |  |  |  |  |  |  |
| Worry | |  | | |  |  | |  |  |  |  |  |  |
| 6 | -7.86 (-15.73, 0.01) | 9.65 (-0.99, 20.30) | | |  |  | | 17.55 |  | **(3.93, 31.16)** |  | **0.012** |  |
| 12 | -4.40 (-12.63, 3.82) | 5.13 (-6.81, 17.07) | | |  |  | | 8.78 |  | (-5.94, 23.51) |  | 0.242 |  |
| F (%) | -2.17 (-11.44, 7.09) | 14.40 (-17.80, 46.62) | | | |  | |  |  |  |  |  |  |
| **Total SF-36** | | | | | | | | | | | |  |  |
| 6 | 0.38 (-8.18, 8.94) | 5.66 (-4.25, 15.57) | | |  |  | | 5.30 |  | (-7.99, 18.60) |  | 0.434 |  |
| 12 | -0.92 (-9.49, 7.63) | 1.80 (-8.74, 12.36) | | |  |  | | 2.25 |  | (-11.47, 15.98) |  | 0.747 |  |
| F (%) | 2.36 (-10.47, 15.19) | -1.17 (-23.96, 21.61) | | | |  | |  |  |  |  |  |  |
| Physical Functioning (PF) | |  | | |  |  | |  |  |  |  |  |  |
| 6 | -5.48 (-18.86, 7.88) | -0.75 (-9.74, 8.23) | | |  |  | | 4.63 |  | (-11.16, 20.44) |  | 0.565 |  |
| 12 | 2.82 (-10.54, 16.20) | 3.39 (-6.20, 12.99) | | |  |  | | 0.62 |  | (-15.65, 16.91) |  | 0.940 |  |
| F (%) | 7.73 (-8.46, 23.94) | 2.50 (-13.34, 18.34) | | | |  | |  |  |  |  |  |  |
| Role - Physical (RP) | |  | | |  |  | |  |  |  |  |  |  |
| 6 | -14.96 (-30.08, 0.14) | 18.18 (-4.28, 40.64) | | | |  | | 33.17 |  | **(5.29, 61.04)** |  | **0.020** |  |
| 12 | -5.94 (-21.06, 9.16) | 10.23 (-13.56, 34.13) | | | |  | | 15.70 |  | (-13.02, 44.43) |  | 0.284 |  |
| F (%) | 2.38 (-17.31, 22.07) | 1.38 (-47.42, 50.20) | | | |  | |  |  |  |  |  |  |
| Bodily Pain (BP) | |  | | |  |  | |  |  |  |  |  |  |
| 6 | -3.01 (-16.19, 10.16) | 10.45 (-5.64, 26.55) | | | |  | | 13.50 |  | (-6.89, 33.91) |  | 0.194 |  |
| 12 | -0.34 (-16.91, 16.23) | 3.61 (-11.88, 19.10) | | | |  | | 3.98 |  | (-16.97, 24.93) |  | 0.710 |  |
| F (%) | -2.42 (-22.69, 17.84) | 12.97 (-11.07, 37.03) | | | |  | |  |  |  |  |  |  |
| General Health (GH) | |  | | |  |  | |  |  |  |  |  |  |
| 6 | 2.85 (-12.46, 18.16) | 13.63 (-1.83, 29.10) | | | |  | | 10.89 |  | (-8.59, 30.39) |  | 0.273 |  |
| 12 | -10.89 (-20.95, -0.82) | 10.92 (-9.12, 30.97) | | | |  | | 21.46 |  | **(1.33, 41.60)** |  | **0.037** |  |
| F (%) | -4.82 (-25.56, 5.92) | 19.48 (-21.18, 60.16) | | | |  | |  |  |  |  |  |  |
| Vitality (VT) | |  | | |  |  | |  |  |  |  |  |  |
| 6 | 7.84 (-6.43, 22.13) | 0.60 (-11.93, 13.15) | | | |  | | -7.24 |  | (-26.53, 12.02) |  | 0.462 |  |
| 12 | -5.95 (-22.51, 10.61) | -8.67 (-24.36, 7.02) | | | |  | | -2.75 |  | (-22.65, 17.14) |  | 0.766 |  |
| F (%) | -1.97 (-39.46, 35.50) | -6.18 (-37.22, 24.84) | | | |  | |  |  |  |  |  |  |
| Social Functioning (SF) | |  | | |  |  | |  |  |  |  |  |  |
| 6 | 11.33 (-8.73, 31.40) | 7.95 (-5.37, 21.28) | | | |  | | -3.41 |  | (-22.96, 16.14) |  | 0.732 |  |
| 12 | 12.79 (0.42, 25.17) | -7.71 (-25.40, 9.97) | | |  |  | | -20.64 |  | **(-40.84, -0.45)** |  | **0.045** |  |
| F (%) | 28.70 (-21.84, 79.26) | -11.25 (-47.59, 25.09) | | | |  | |  |  |  |  |  |  |
| Role-Emotional (RE) | |  | | |  |  | |  |  |  |  |  |  |
| 6 | 11.79 (-17.07, 40.65) | 3.03 (-15.56, 21.62) | | | |  | | -9.05 |  | (-43.22, 25.11) |  | 0.604 |  |
| 12 | 12.30 (-15.02, 39.63) | -6.15 (-32.17, 19.86) | | | |  | | -15.51 |  | (-50.73, 19.70) |  | 0.388 |  |
| F (%) | 0.01 (-34.88, 34.88) | -10.41 (-53.37, 32.53) | | | |  | |  |  |  |  |  |  |
| Mental Health (MH) | |  | | |  |  | |  |  |  |  |  |  |
| 6 | 3.31 (-6.94, 13.56) | 1.54 (-10.58, 13.67) | | | |  | | -1.81 |  | (-17.23, 13.60) |  | 0.817 |  |
| 12 | -2.12 (-14.97, 10.73) | -4.82 (-16.76, 7.11) | | |  |  | | -3.15 |  | (-19.11, 12.79) |  | 0.698 |  |
| F (%) | 9.43 (-33.24, 52.11) | 4.16 (-32.19, 40.52) | | | |  | |  |  |  |  |  |  |
| Section A: Physical Health | |  | | |  |  | |  |  |  |  |  |  |
| 6 | -2.55 (-12.72, 7.61) | 8.42 (-4.93, 21.78) | | |  |  | | 11.04 |  | (-3.51, 25.59) |  | 0.137 |  |
| 12 | -4.65 (-12.56, 3.26) | 4.08 (-9.00, 17.14) | | |  |  | | 8.21 |  | (-6.80, 23.22) |  | 0.284 |  |
| F (%) | -0.99 (-16.58, 14.59) | 5.15 (-24.61, 34.92) | | | |  | |  |  |  |  |  |  |
| Section B: Mental Health | |  | | |  |  | |  |  |  |  |  |  |
| 6 | 7.52 (-6.19, 21.24) | 5.35 (-5.46, 16.17) | | |  |  | | -2.17 |  | (-18.38, 14.04) |  | 0.793 |  |
| 12 | 0.68 (-9.64, 11.01) | -3.10 (-17.96, 11.74) | | | |  | | -4.06 |  | (-20.81, 12.68) |  | 0.634 |  |
| F (%) | 2.04 (-19.39, 22.48) | -8.71 (-33.58, 16.14) | | | |  | |  |  |  |  |  |  |
| ^*^ CIs and P-values were adjusted using a Generalized Estimating Equation (GEE) by Body Mass Index (BMI), age, and sex as confounders.  MSCs: Mesenchymal stem cells | | | | | | | | | | | | |  |
| CI: Confidence interval  MD: Mean difference  Significant P-values are shown in **bold** type | | | | DQOL: Diabetes specific Quality of Life  ^a^(\|Baseline – month 12\| / Baseline) *100  SF-36: 36-Item Short Form Survey | | | | | | | | |  |
|  | | | | | | | | | | | | |  |

| **Table S8.** Comparison of quality of life (QOL) questionnaire scores in 12 months of follow-up between the early (Early Tx) and late (Late Tx) transplantation groups | | | | | | | | | |  |
| --- | --- | --- | --- | --- | --- | --- | --- | --- | --- | --- |
|  | Test of within-group effects^*^  (mean change from baseline) | | |  | Test of between-group effects^*^  (mean change from reference group) | | | | |  |
| **Outcomes/ Time (month)** | **Early Tx (n=11)** | **Late Tx (n=9)** | |  | **Late Tx vs. Early Tx** | | | | | |
|  | **Mean (95% CI)** | **Mean (95% CI)** | |  | **MD** |  | **95% CI** |  | **P-value** |  |
| **Total DQOL** | | | |  | | | |  |  |  |
| 6 | -0.99 (-6.54, 4.56) | 4.53 (-4.20, 13.27) | |  | 5.93 |  | (-4.01, 15.89) |  | 0.243 |  |
| 12 | -6.22 (-12.43, -0.02) | 6.01 (-2.72, 14.76) | |  | 11.83 |  | **(1.40, 22.26)** |  | **0.026** |  |
| F^a^ (%) | -7.27 (-17.60, 3.04) | 8.64 (-12.39, 29.68) | |  |  |  |  |  |  |  |
| Satisfaction | |  | |  |  |  |  |  |  |  |
| 6 | -1.43 (-9.18, 6.31) | 2.25 (-10.22, 14.74) | |  | 4.45 |  | (-9.62, 18.54) |  | 0.535 |  |
| 12 | -7.51 (-16.17, 1.15) | 3.66 (-8.82, 16.14) | |  | 10.30 |  | (-4.44, 25.06) |  | 0.171 |  |
| F (%) | -5.87 (-27.43, 15.68) | 7.87 (-22.46, 38.20) | |  |  |  |  |  |  |  |
| Impact | |  | |  |  |  |  |  |  |  |
| 6 | -1.01 (-6.88, 4.73) | 1.69 (-7.67, 11.05) | |  | 3.34 |  | (-7.22, 13.91) |  | 0.535 |  |
| 12 | -5.63 (-12.13, 0.86) | 2.74 (-6.61, 12.11) | |  | 7.72 |  | (-3.33, 18.79) |  | 0.171 |  |
| F (%) | -8.82 (-22.63, 4.97) | 12.96 (-6.60, 32.52) | |  |  |  |  |  |  |  |
| Worry | |  | |  |  |  |  |  |  |  |
| 6 | 5.88 (-1.72, 13.49) | -0.47 (-11.23, 10.27) | |  | -5.75 |  | (-18.59, 7.08) |  | 0.380 |  |
| 12 | -1.76 (-10.31, 6.78) | 1.75 (-8.99, 12.51) | |  | 3.41 |  | (-10.07, 16.90) |  | 0.620 |  |
| F (%) | 13.24 (-11.24, 37.73) | 4.59 (-17.12, 26.31) | |  |  |  |  |  |  |  |
| **Total SF-36** | | | |  |  | | | |  |  |
| 6 | 5.66 (-5.57, 16.89) | 8.51 (-1.91, 18.94) | |  | 3.00 |  | (-11.21, 17.21) |  | 0.679 |  |
| 12 | 1.80 (-10.26, 13.88) | 6.56 (-3.15, 16.29) | |  | 4.70 |  | (-9.88, 19.30) |  | 0.527 |  |
| F (%) | 8.42 (-20.60, 37.46) | 9.73 (-7.99, 27.45) | |  |  |  |  |  |  |  |
| Physical Functioning (PF) | |  | |  |  |  |  |  |  |  |
| 6 | -0.75 (-10.60, 9.08) | 6.15 (-0.62, 12.93) | |  | 6.84 |  | (-5.46, 19.16) |  | 0.276 |  |
| 12 | 3.39 (-6.86, 13.65) | 6.15 (-2.89, 15.21) | |  | 2.71 |  | (-9.94, 15.37) |  | 0.674 |  |
| F (%) | 6.38 (-10.39, 23.16) | 8.25 (-7.54, 24.04) | |  |  |  |  |  |  |  |
| Role- Physical (RP) | |  | |  |  |  |  |  |  |  |
| 6 | 18.18 (-3.82, 40.18) | 4.62 (-7.21, 16.46) | |  | -13.43 |  | (-41.72, 14.86) |  | 0.352 |  |
| 12 | 10.23 (-13.75, 34.23) | -2.32 (-14.64, 9.98) | |  | -12.78 |  | (-41.82, 16.25) |  | 0.388 |  |
| F (%) | 38.54 (-48.94, 126.03) | -2.38 (-18.32, 13.56) | |  |  |  |  |  |  |  |
| Bodily Pain (BP) | |  | |  |  |  |  |  |  |  |
| 6 | 10.45 (-5.64, 26.55) | 4.33 (-8.55, 17.22) | |  | -6.60 |  | (-27.91, 14.71) |  | 0.544 |  |
| 12 | 3.61 (-11.88, 19.10) | 2.84 (-15.85, 21.54) | |  | -1.75 |  | (-23.64, 20.13) |  | 0.875 |  |
| F (%) | 22.58 (-24.55, 41.66) | 8.55 (-24.55, 41.66) | |  |  |  |  |  |  |  |
| General Health (GH) | |  | |  |  |  |  |  |  |  |
| 6 | 13.63 (-1.83, 29.10) | 5.67 (-10.93, 22.27) | |  | -8.03 |  | (-29.61, 13.55) |  | 0.466 |  |
| 12 | 10.92 (-9.12, 30.97) | 4.52 (-10.56, 19.61) | |  | -5.97 |  | (-28.18, 16.23) |  | 0.598 |  |
| F (%) | 48.54 (-43.29, 140.38) | 4.71 (-4.39, 33.83) | |  |  |  |  |  |  |  |
| Vitality (VT) | |  | |  |  |  |  |  |  |  |
| 6 | 0.60 (-11.93, 13.15) | 10.53 (-3.35, 24.43) | |  | 10.51 |  | (-8.49, 29.52) |  | 0.278 |  |
| 12 | -8.67 (-24.36, 7.02) | 2.88 (-13.38, 19.16) | |  | 11.53 |  | (-7.99, 31.07) |  | 0.247 |  |
| F (%) | -2.27 (-34.48, 29.93) | 10.71 (-21.75, 43.18) | |  |  |  |  |  |  |  |
| Social Functioning (SF) | |  | |  |  |  |  |  |  |  |
| 6 | 7.95 (-5.37, 21.28) | 20.22 (-0.35, 40.80) | |  | 12.20 |  | (-8.11, 32.52) |  | 0.239 |  |
| 12 | -7.71 (-25.40, 9.97) | 16.21 (-1.40, 33.84) | |  | 23.93 |  | **(3.03, 44.83)** |  | **0.025** |  |
| F (%) | -5.64 (-33.65, 22.35) | 57.73 (-21.61, 137.08) | |  |  |  |  |  |  |  |
| Role-Emotional (RE) | |  | |  |  |  |  |  |  |  |
| 6 | 0.03 (-15.56, 21.62) | 10.58 (-23.07, 44.24) | |  | 9.17 |  | (-23.86, 42.21) |  | 0.586 |  |
| 12 | -6.15 (-32.17, 19.86) | 25.79 (6.08, 45.50) | |  | 31.13 |  | (-2.82, 65.09) |  | 0.072 |  |
| F (%) | -10.41 (-53.95, 33.12) | 16.66 (-6.10, 39.43) | |  |  |  |  |  |  |  |
| Mental Health (MH) | |  | |  |  |  |  |  |  |  |
| 6 | 1.54 (-10.58, 13.67) | 12.95 (-5.46, 31.37) | |  | 11.65 |  | (-5.66, 28.97) |  | 0.187 |  |
| 12 | -4.82 (-16.76, 7.11) | 1.39 (-7.43, 10.22) | |  | 6.05 |  | (-11.77, 23.88) |  | 0.505 |  |
| F (%) | 4.23 (-27.39, 35.85) | -0.08 (-10.40, 10.23) | |  |  |  |  |  |  |  |
| Section A: Physical Health | |  | |  |  |  |  |  |  |  |
| 6 | 8.42 (-4.93, 1.78) | 5.79 (-3.67, 15.26) | |  | -2.44 |  | (-18.70, 13.80) |  | 0.768 |  |
| 12 | 4.07 (-9.00, 17.14) | 2.72 (-9.24, 14.69) | |  | -1.31 |  | (-17.98, 15.35) |  | 0.877 |  |
| F (%) | 25.70 (-19.09, 70.51) | 5.97 (-15.91, 27.85) | |  |  |  |  |  |  |  |
| Section B: Mental Health | |  | |  |  |  |  |  |  |  |
| 6 | 5.34 (-5.46, 16.17) | 12.45 (-3.87, 28.77) | |  | 7.11 |  | (-9.58, 23.81) |  | 0.404 |  |
| 12 | -3.10 (-17.96, 11.74) | 10.25 (-1.72, 22.24) | |  | 13.35 |  | (-3.82, 30.53) |  | 0.128 |  |
| F (%) | 6.42 (-34.04, 46.89) | 17.45 (-14.39, 49.30) | |  |  |  |  |  |  |  |
| ^*^ CIs and P-values were adjusted using a generalized estimating equation (GEE) by body mass index (BMI), age, and sex as confounders. | | | | | | | | | |  |
| Tx: Transplantation  CI: Confidence interval  MD: Mean difference  Significant P-values are shown in **bold** type | | | DQOL: Diabetes specific Quality of Life  ^a^ (\|Baseline – Month 12\| / Baseline) *100  SF-36: 36-Item Short Form Survey | | | | | | |  |

| **Table S9.** Comparison of metabolic indices in 12 months of follow-up between early versus late transplantation of MSCs | | | | | | | | | |
| --- | --- | --- | --- | --- | --- | --- | --- | --- | --- |
|  | Test of Within-group effects^*^  (mean change from baseline) | | |  | Test of between-group effects^*^  (mean change from reference group) | | | | |
| **Outcomes/ Time (month)** | **Early Tx (n=11)** | **Late Tx (n=9)** | |  | **Late Tx vs. Early Tx** | | | | |
|  | **Mean (95% CI)** | **Mean (95% CI)** | |  | **MD** |  | **95% CI** |  | **P-value** |
| FBS (mg/dl) | | | | | | | | | |
| 3 | 88.72 (17.30, 160.14) | 32.88 (-15.03, 80.81) | |  | -55.86 | | (-46.34, 34.66) |  | 0.227 |
| 6 | 84.63 (13.21, 156.05) | 35.50 (16.04, 87.05) | |  | -47.02 | | (-141.77, 47.73) | | 0.331 |
| 9 | 74.88 (-3.99, 153.76) | -7.26 (-76.52, 61.98) | |  | -82.96 | | (-202.51, 36.58) | | 0.174 |
| 12 | 87.18 (15.76, 158.60) | 41.92 (-7.62, 91.47) | |  | -42.36 | | (-34.79, 50.06) |  | 0.369 |
| F^a^ (%) | 96.30 (0.43, 192.18) | 38.08 (-29.37, 105.54) | |  |  |  |  |  |  |
| 2hpp (mg/dl) | | | | | | | | | |
| 3 | 59.46 (-52.77, 171.70) | 67.50 (-21.58, 156.59) | |  | 9.11 | | (-139.06, 157.30) | | 0.904 |
| 6 | 100.55 (-11.68, 212.80) | 65.12 (-27.39, 157.63) | |  | -25.56 | | (-177.47, 126.33) | | 0.741 |
| 12 | 94.37 (-17.86, 206.61) | 30.10 (-58.98, 119.20) | |  | -59.12 | | (-207.41, 89.15) | | 0.434 |
| F (%) | 112.52 (-12.56, 237.61) | 23.39 (-23.04, 69.83) | |  |  |  |  |  |  |
| HbA1c (%) | | | | | | | | | |
| 3 | 0.02 (-0.89, 0.93) | 2.96 (2.05, 3.86) | |  | 2.95 |  | **(1.62, 4.28)** |  | **<0.001** |
| 6 | 0.27 (-0.64, 1.18) | 1.99 (1.04, 2.94) | |  | 1.69 |  | **(0.32, 3.06)** |  | **0.015** |
| 9 | -0.01 (-1.06, 1.06) | 0.94 (-0.35, 2.25) | |  | 0.86 |  | (-0.89, 2.63) |  | 0.335 |
| 12 | -0.46 (-1.41, 0.47) | 0.95 (0.04, 1.86) | |  | 1.40 |  | **(0.06, 2.76)** |  | **0.041** |
| F (%) | -2.16 (-4.96, 10.64) | 12.75 (-6.98, 32.48) | |  |  |  |  |  |  |
| C-peptide (ng/ml) | | | | | | | | | |
| 3 | -0.15 (-0.34, 0.03) | -0.74 (-1.10, -0.37) | |  | -0.58 |  | **(-0.96, -0.20)** |  | **0.003** |
| 6 | -0.38 (-0.57, -0.19) | -0.82 (-1.21, -0.42) | |  | -0.46 |  | **(-0.85, -0.06)** |  | **0.023** |
| 9 | -0.39 (-0.60, -0.19) | -0.89 (-1.37, -0.42) | |  | -0.56 |  | **(-1.02, -1.10)** |  | **0.016** |
| 12 | -0.41 (-0.60, -0.22) | -0.63 (-1.01, -0.25) | |  | -0.22 |  | (-0.61, 0.16) |  | 0.249 |
| F^b^ (6) (%) | -36.33 (-84.04, 11.37) | -77.23 (-111.27, -43.11) | | |  |  |  |  |  |
| F^c^ (9) (%) | -34.06 (-90.14, 22.02) | -82.96 (-115.51, -50.40) | | |  |  |  |  |  |
| F^a^ (12) (%) | -46.49 (-86.15, -6.84) | -69.05 (-98.32, -39.77) | |  |  |  |  |  |  |
| EI (I.U./kg/day) | | | | | | | | | |
| 6 | 0.02 (-0.23, 0.28) | 0.24 (0.03, 0.46) | |  | 0.21 |  | (-0.14, 0.57) |  | 0.245 |
| 12 | 0.24 (-0.02, 0.51) | 0.23 (0.03, 0.42) | |  | -0.01 |  | (-0.35, 0.33) |  | 0.954 |
| F (%) | 76.10 (-9.66, 161.87) | 48.06 (-18.88, 115.01) | |  |  |  |  |  |  |
| LI (mmol/l^2^/h.week^-1^) | | | | | | | | | |
| 6 | 55.26 (15.26, 95.27) | 10.27 (-29.00, 49.55) | |  | -46.17 | | (-104.34, 11.98) | | 0.120 |
| 12 | 10.05 (-31.45, 51.56) | -2.55 (-37.83, 32.72) | |  | -13.07 | | (-68.47, 42.32) |  | 0.644 |
| F (%) | 336.94 (-598.87, 1272.76) | 1714.12 (-2238.98, 5667.23) | |  |  |  |  |  |  |
| ^*^ CIs and P-values were adjusted using a generalized estimating equation (GEE) by body mass index (BMI), age, and sex as confounders. | | | | | | | | | |
| MSCs: Mesenchymal stem cells  Tx: Transplantation  CI: Confidence interval  MD: Mean difference  HbA1c: Glycated hemoglobin  Significant P-values are shown in **bold** type.  ^a^ (\|Baseline – Month 12\| / Baseline) *100 | | | ^b^ (\|Baseline – Month 6\| / Baseline) *100  ^c^ (\|Baseline – Month 9\| / Baseline) *100  EI: Exogenous insulin  LI: Lability index  FBS: Fasting blood sugar  2hpp: Two-hour postprandial | | | | | | |

| **Table S10.** Comparison of immunologic indices in 12 months of follow-up for the early (Early Tx) and late (Late Tx) transplantation groups | | | | | | | |
| --- | --- | --- | --- | --- | --- | --- | --- |
|  | Test of within-group effects^*^  (mean change from baseline) | | |  | Test of between-group effects^*^  (mean change from reference group) | | |
| **Outcomes/**  **Time (month)** | **Early Tx (n=11)** | **Late Tx (n=9)** | |  | **Late Tx vs. Early Tx** | | |
|  | **Mean (95% CI)** | **Mean (95% CI)** | |  | **MD** | **95% CI** | **P-value** |
| **Pro-inflammatory** | | | | | | | |
| IL-6 (ng/ l) | | | | | | | |
| 6 | -3.90 (-34.39, 26.57) | 20.59 (-9.59, 50.79) | |  | 23.74 | (-21.12, 68.61) | 0.300 |
| 12 | -16.75 (-47.24, 13.72) | -19.72 (-47.40, 7.95) | |  | -3.37 | (-45.68, 39.00) | 0.877 |
| F^a^ (%) | -6.37 (-37.77, 25.02) | -13.60 (-65.48, 38.27) | |  |  |  |  |
| TNF-α (ng/l) | | | | | | | |
| 6 | -6.17 (-34.22, 21.87) | 59.15 (29.62, 88.68) | |  | 65.80 | **(23.53, 108.07)** | **0.002** |
| 12 | -5.89 (-33.94, 22.18) | 38.64 (11.92, 65.35) | |  | 44.68 | **(4.81, 84.55)** | **0.028** |
| F (%) | 6.55 (-14.40, 27.51) | 67.42 (13.51, 121.32) | |  |  |  |  |
| **Anti-inflammatory** | |  | |  |  |  |  |
| IL-4 (pg/ml) | | | | | | | |
| 6 | 52.47 (-146.52, 258.46) | -235.58 (-327.65, -143.51) | | -290.47 | | **(-544.41, -36.53)** | **0.025** |
| 12 | 161.78 (-37.21, 360.78) | -233.67 (-317.32, -150.01) | | -392.88 | | **(-631.81, -153.96)** | **0.001** |
| F (%) | 61.56 (-25.15, 148.28) | -75.54 (-94.99, -56.09) | |  |  |  |  |
| IL-10 (pg/ml) | | | | | | | |
| 6 | 3.99 (-72.46, 80.46) | -353.46 (-574.73, -32.20) | | -387.24 | | **(-588.20, -186.27)** | **<0.001** |
| 12 | 7.91 (-68.54, 84.38) | -366.66 (-568.63, -64.69) | | -387.20 | | **(-575.89, -198.51)** | **<0.001** |
| F (%) | 12.81 (-38.45, 64.09) | -83.14 (-98.38, -67.90) | |  |  |  |  |
| TGF-β1 (pg/ml) | | | | | | | |
| 6 | 43.01 (-531.44, 617.46) | -716.66 (-970.95, -462.37) | | -775.93 | | **(-1510.37, -41.49)** | **0.038** |
| 12 | 152.14 (-422.31, 726.59) | -709.32 (-943.64, -475.00) | | -857.35 | | **(-1552.19, -162.51)** | **0.016** |
| F (%) | 119.43 (-174.10, 412.97) | -83.31 (-100.65, -65.98) | |  |  |  |  |
| ^*^ CIs and P-values were adjusted using a generalized estimating equation (GEE) with body mass index (BMI), age, and sex as confounders. | | | | | | | |
| Tx: Transplantation  CI: Confidence interval  MD: Mean difference  Significant P-values are shown in **bold** type.  IL-6: Interleukin 6 | | | ^a^ (\|Baseline – Month 12\| / Baseline) *100  TNF-α: Tumor necrosis factor-alpha  IL-4: Interleukin 4  IL-10: Interleukin 10  TGF-β1: Transforming growth factor-beta 1 | | | | |


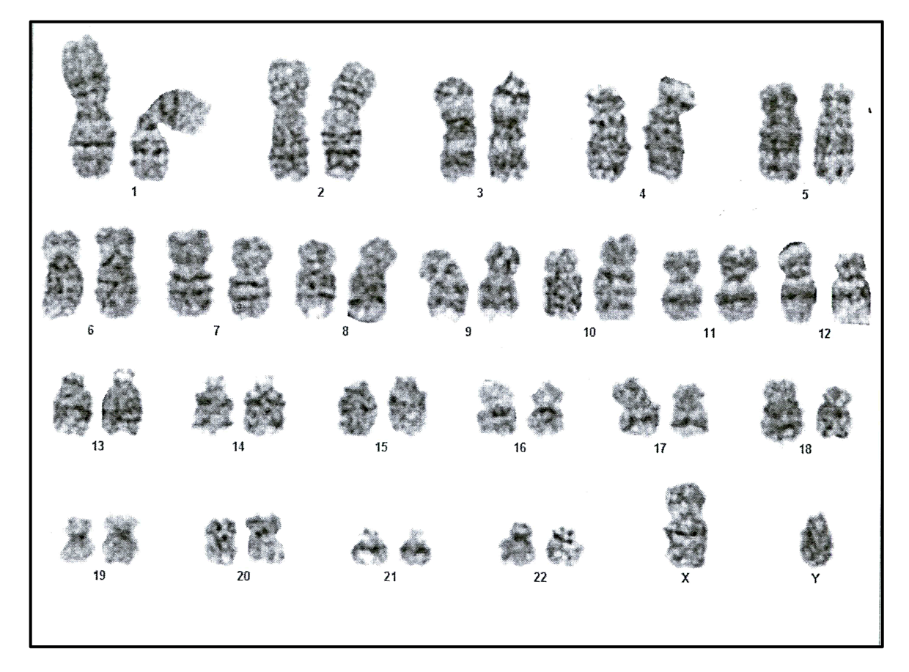


**Figure S1.** **Sample of cytogenetic report of MSCs.** Karyotyping was performed on passage 3 expanded mesenchymal stem cells. The results showed a normal male karyotype (46, XY) in this sample.


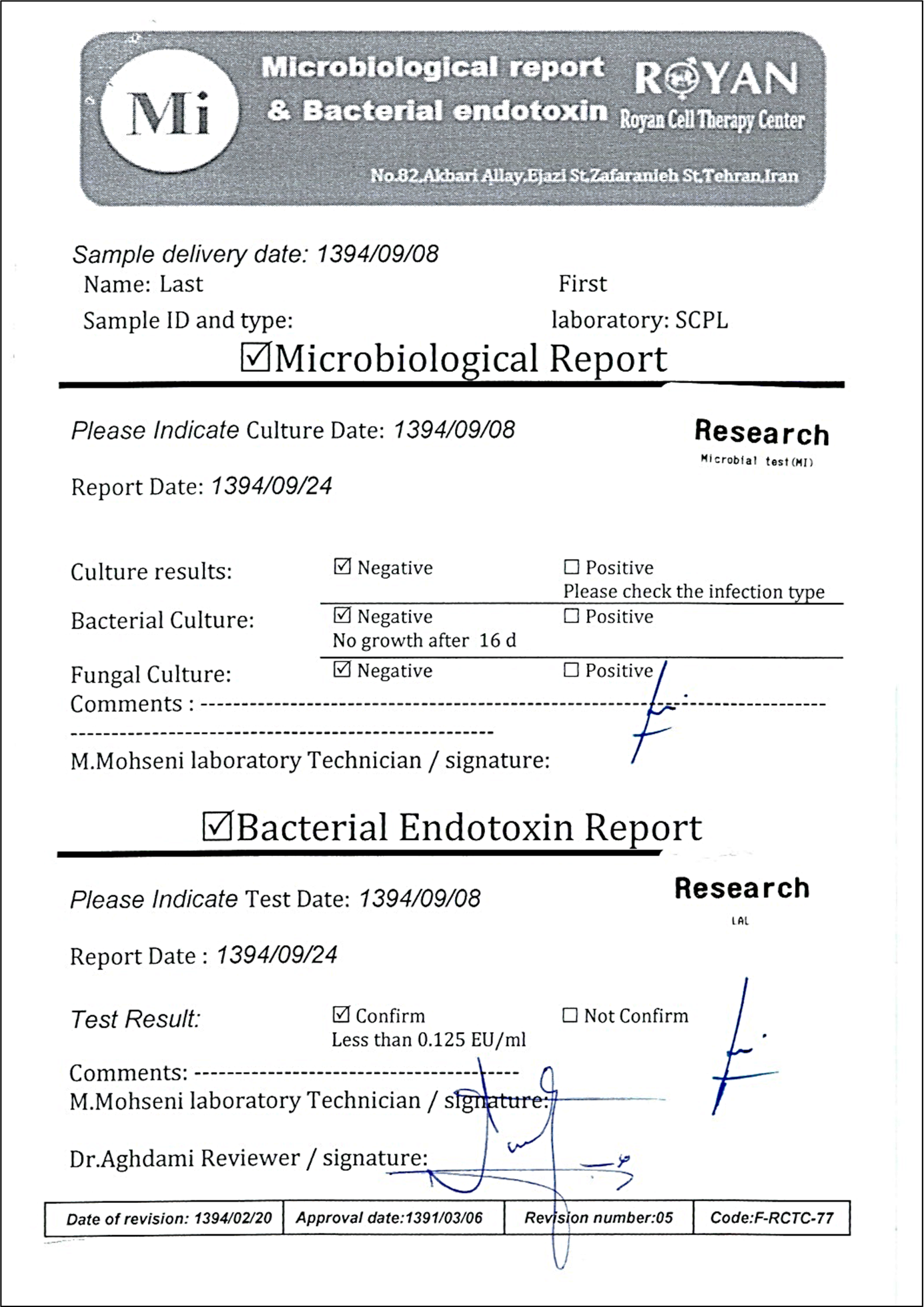


**Figure S2. Sample of microbiological and bacterial endotoxin report.** Bacterial and fungal culture as well as bacterial endotoxin level assessment were performed in the quality control department to confirm the safety of prepared Mesenchymal Stem Cells prior to transplantation. Patient information was removed to protect their privacy and confidentiality.


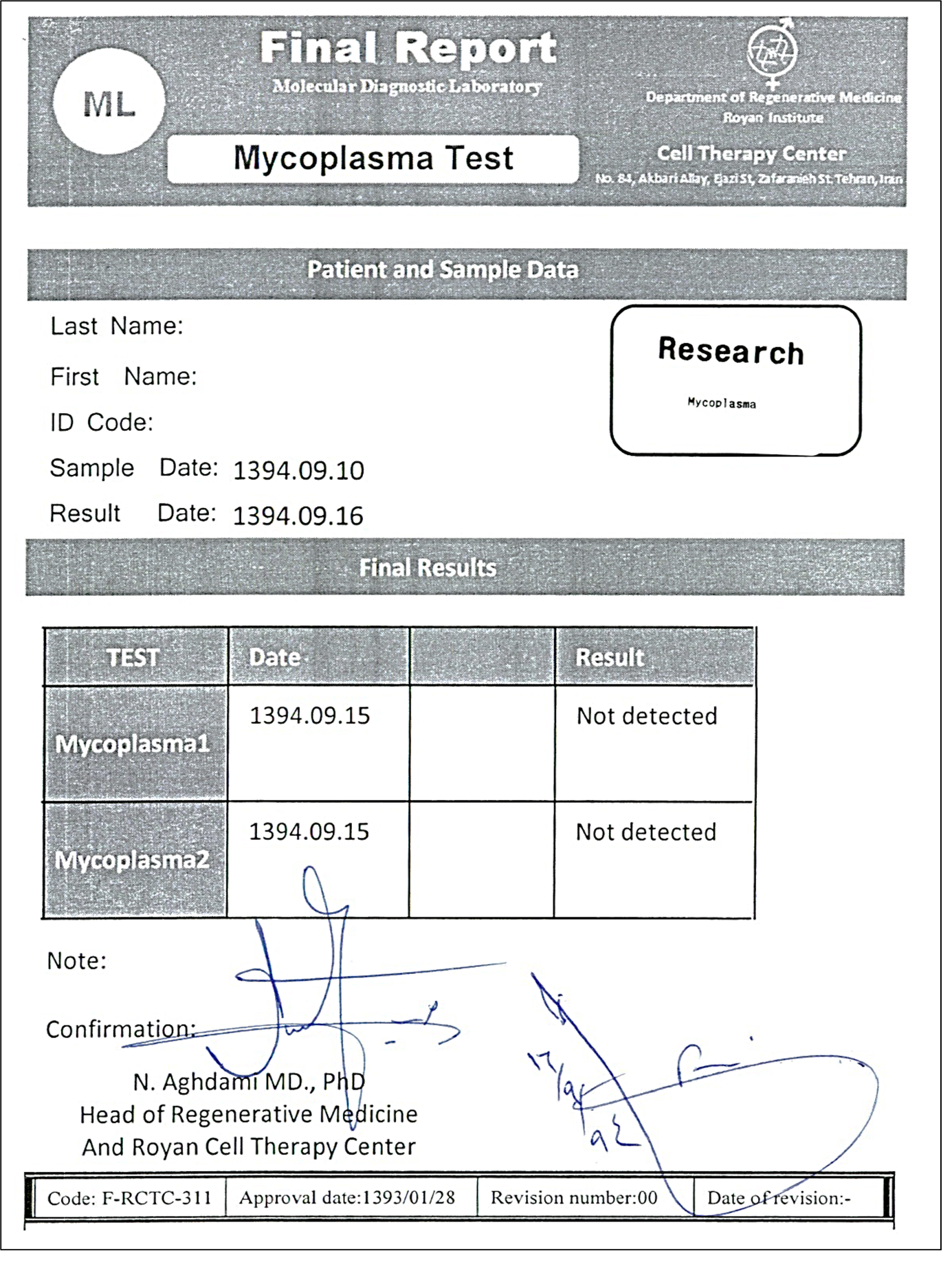


**Figure S3. Sample of mycoplasma report.** MSCs preparations were checked for the presence of Mycoplasma. Patient information was removed to protect their privacy and confidentiality.


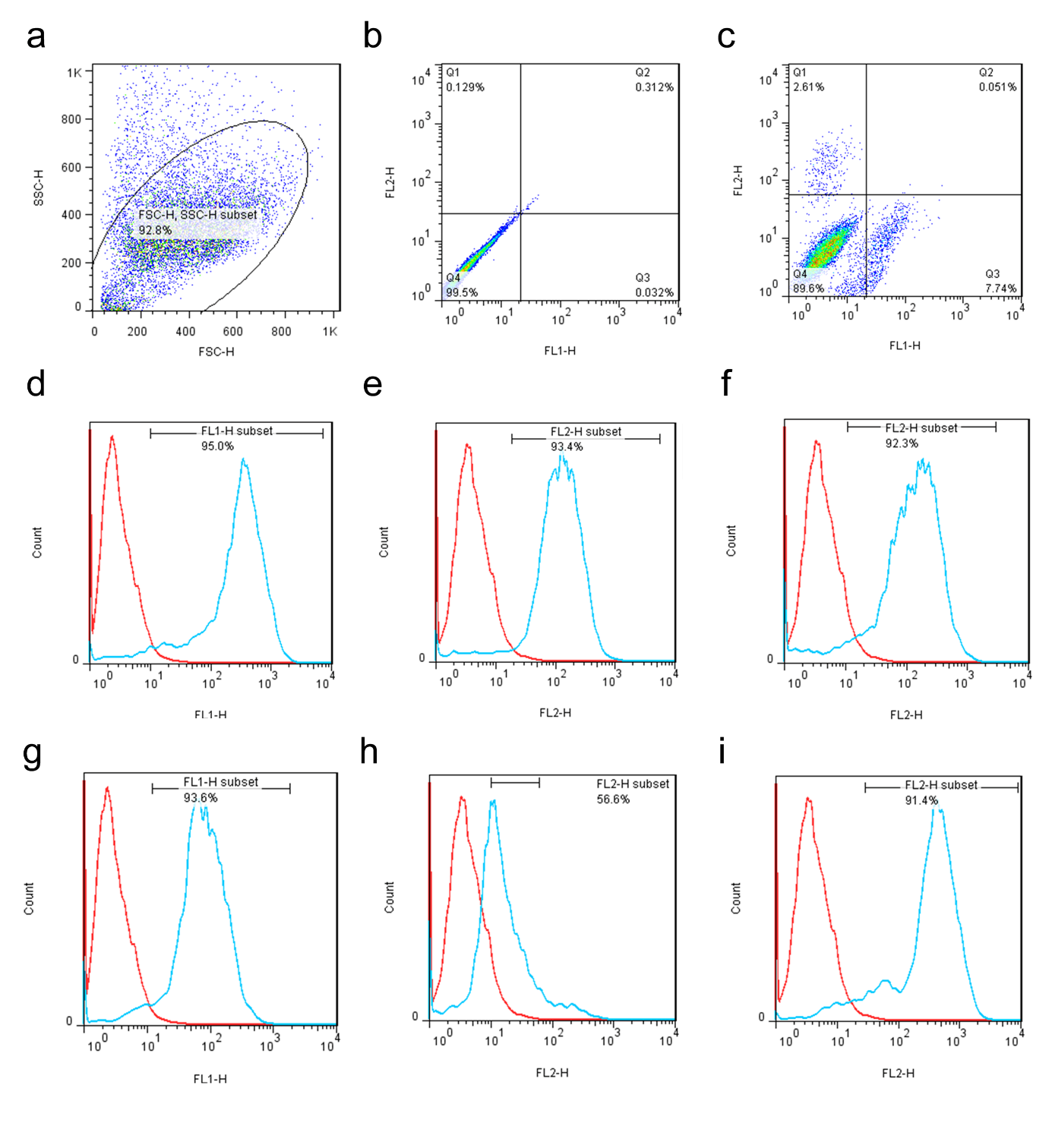


**Figure S4. Flow Cytometric Analysis of Mesenchymal Stem Cells.** The MSCs were assessed by flow cytometry for their ability to express CD105, CD90, CD44, CD73, and lack of CD34 and CD45 expression. **a.** G1-FITC/G1-PE, **b.** G1-FITC/G1-PE, **c.** CD45-FITC/CD34-PE, **d.** CD90-FITC, **e.** CD105-PE, **f.** CD73-PE, **g.** CD44-FITC, **h.** CD11b-PE, **i.** CD29-PE.


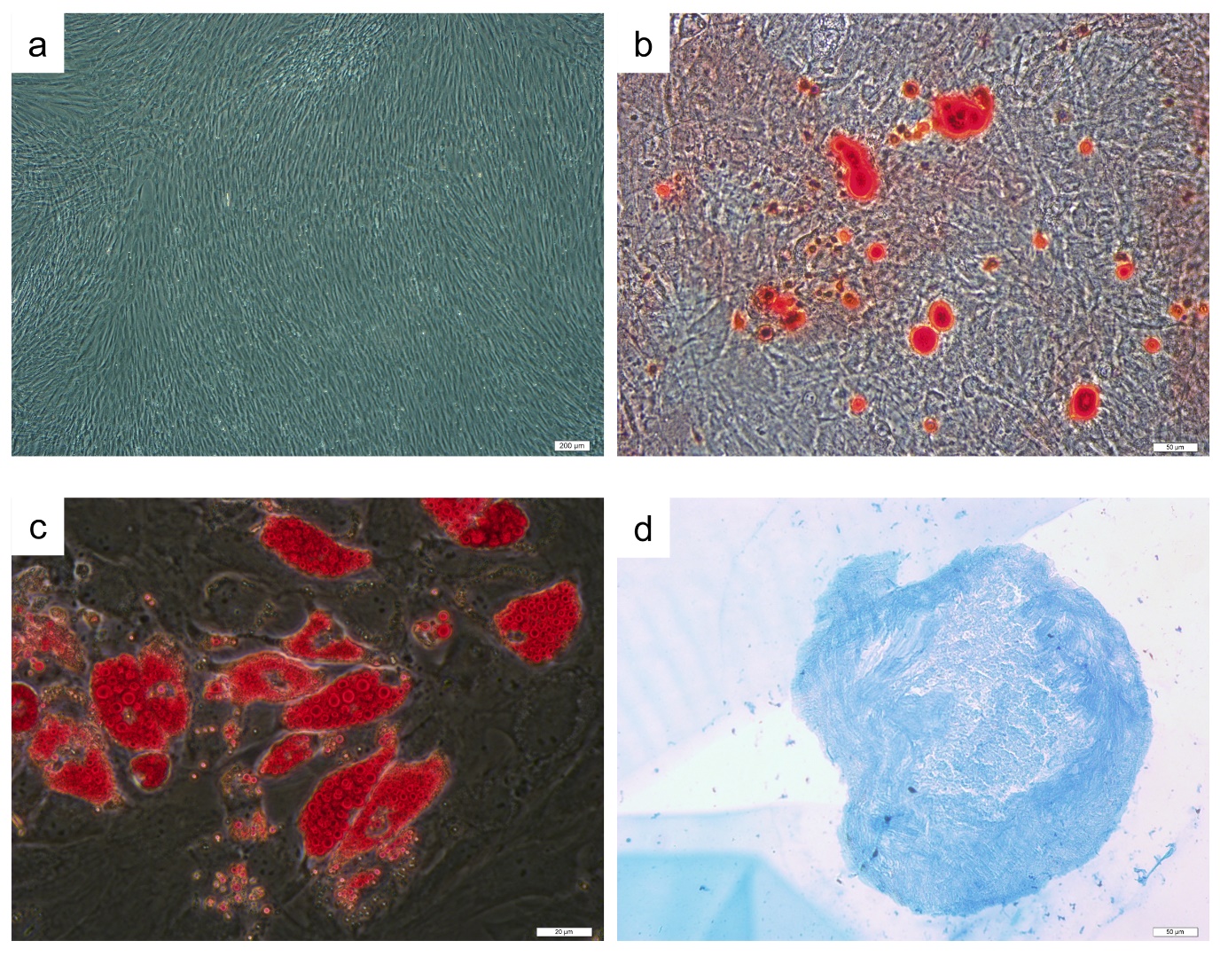


**Figure S5. Mesenchymal Stem Cells (MSCs) morphology and differentiation potential.** MSCs morphology and their ability to differentiate into different lineages were checked. **a.** Morphology; MSCs were differentiated to **b.** osteocytes, **c.** adipocytes, and **d.** chondrocytes. The scale bar is 200 μm in figure a, 50 μm in b & d, and 20 μm in figure c.
